# Impact of social media platforms excessive use on students performance in higher education institutions: A mathematical model approach

**DOI:** 10.1371/journal.pone.0359301

**Published:** 2026-10-01

**Authors:** Komi Afassinou, Narcisse Roland Loufouma Makala, Ousmane Koutou, Richard O. Ocaya, Maba Boniface Matadi

**Affiliations:** 1 Department of Mathematics and Applied Mathematics, University of the Free State, QwaQwa Campus, Phuthaditjhaba, South Africa; 2 Centre Universitaire Polytechnique de Kaya, Université Joseph KI-Zerbo, Laboratoire de Mathématiques et Informatique, Ouagadougou, Burkina Faso; 3 Department of Physics, University of the Free State, QwaQwa Campus, Phuthaditjhaba, South Africa; 4 Department of Mathematical Sciences, University of Zululand, KwaDlangezwa, South Africa; Beijing University of Technology, CHINA

## Abstract

This article develops a compartmental mathematical model to investigate the impact of excessive social media use on the academic performance of higher education students. Students who are heavily subscribed are classified ‘infected or addicted’ individuals. Their influence on the other naive non-subscribers leading them to become one and by self-curiosity are defined as direct and indirect transmission routes, respectively. The model incorporates two primary control interventions: early awareness programs and continuous regulation of accessibility. Qualitative and numerical analyses of the model dynamics are thus carried out investigating the effectiveness and the benefits of these control measures. The results suggest that a combined strategy, implementing both control interventions at optimal efficacy levels, significantly reduces the ‘addiction’ rate, thereby fostering a healthier academic environment and promising a prospective academic performance from the students.

## Introduction

Social media platforms such as TikTok, Facebook, Instagram, and others reign supreme in the modern digital landscapes, captivating the attention of the youth worldwide with their addictive contents (videos, photos, vlogs, live-streams) and with their possibilities of one to express his/her personal opinions (likes, shares, comments, emoticons, and so on). According to Pew Research Center report in [1], approximately all teens (97%) are now social media users, of whom 88% saying they use it daily. In 2022, a report in [2] highlighted that teens spend an average of 7.5 hours per day on screens with social media accounting for nearly a third of that time. This may result from the rapid growth of the number of mobile devices incorporated with different social networking applications that flowed into the digital world in the last decade [3]. The number of subscribers on each of these social media applications have grown exponentially and contents are uploaded millions per second. As of 2024, Facebook is the largest social media platform with 3.07 billion monthly active users worldwide, followed by YouTube (2.5 billion), WhatsApp, Instagram, TikTok, WeChat, etc..[4].. This pervasive usage of these platforms have caused so many problems and raised concerns across different sectors:

In health sectors, professionals have identified a strong link between excessive social media use and mental health issues, including anxiety, depression, and loneliness. Within educational sectors, these entertainment channels constitute a major source of distraction among students, leading to procrastination of their studying time and sleep deprivation, therefore compromising their study flow and ultimately hindering focus and academic achievement [5]. Governments and higher education institutions have a grieving concern about the national financial support (loans, bursaries, and other grants) wasted due to poor student academic performances linked to this issue.

The challenge of excessive social media use among university students can be identified to drug abuse or illicit substance addiction, as frequent use of social media releases dopamine, a chemical substance in the brain, associated with pleasure, reward processing and habit formation [6]. The multi-faceted damages caused by this phenomenon demand urgent and evidence-based interventions to preserve the integrity of the academic environment. While the roles of educators and policy-makers are critical, mathematical modelling offers a unique quantitative framework to simulate the efficacy of potential solutions.

So far, the majority of research documented in this field has relied on longitudinal studies and qualitative reviews [7–10]. Nevertheless, Ali *et al.*, in [11], successfully mapped social media use as a homogeneous contagious trend via a mathematical model. Similarly, Alemneh and Alemu [12] developed a compartmental model incorporating social media awareness and addiction control strategies. Some few compartmental mathematical models including [13,14] were also proposed in this regard. However, the authors in these pioneer social media addiction compartmental models widely focused their analysis on single-route peer-to-peer transmission, while overlook the dual nature of transmission, specifically the distinction between peer-to-peer influence and direct student-to-media contact. This underscores a significant gap remaining to be addressed in compartmental mathematical models to social media addiction studies. This article addresses this gap by developing a model that partitions the student population into distinct classes based on their level of platform engagement and susceptibility. By explicitly branching the susceptible and infected channels, the model thus builds upon existing literature by isolating how institutional intervention interacts with direct exposure to additive social media contents.

The primary objective of this study is to investigate the dynamics of control interventions–specifically early awareness programs and accessibility regulation–and their impact on student academic outcomes. By conducting qualitative stability analysis and numerical simulations, this study aims to provide a theoretical cornerstone for institutional decision-making regarding digital wellness policies.

## Model formulation

A total population of $N_{s}(t)$ students at a time *t*, who are assumed completely susceptible, are mutually co-habiting with a certain number $N_{i}(t)$ of so-called infected students; that is, the active subscribers *I*(*t*) and the addicted subscribers *A*(*t*). These students are very active on *n* distinct social media, say, ${ℳ}_{1}$, ${ℳ}_{2}$, ${ℳ}_{3}$, ..., ${ℳ}_{n}$, available within a given educational institution. These social media platforms contain *M*_1_(*t*), *M*_2_(*t*), *M*_3_(*t*), ..., $M_{n}(t)$ amounts of additive contents (videos, photos, vlogs, live streams, etc...), at time *t*, respectively. The model then partitions the susceptible students population $N_{s}(t)$ into two distinct compartments: *S*(*t*) and $S_{p}(t)$; respectively composed of purely susceptible students *S*(*t*), of whom a proportion γ is enrolled in counselling and awareness programs about the harmful impact of excessive use of social platforms on their academic performances; these later form the compartment $S_{p}(t)$. Thus,


$$\begin{array}{c} N_{s}(t)=S(t)+S_{p}(t). \end{array}$$ (1)

The remaining proportion (1−γ)S, not aware, is then exposed to the risk of excessive social media use and become infected under the attraction of the force of infection λ. Upon being infected, they are classified into *I*(*t*) compartment. Moreover, one may admit that some students from $S_{p}(t)$ compartment, despite the awareness programs priorly received, may fail to obey and become infected. Thus, a proportion $\left(1-\varepsilon_{p}\right)$ of $S_{p}(t)$ is assumed becoming infected due to the pre-exposure awareness failure and thus generate a compartment denoted by $I_{p}(t)$. The parameter $\varepsilon_{p}\in (0,1)$ accounts for the efficacy level of the pre-exposure awareness programs implemented within the institution. Upon developing obsessive interest in social media contents, infected students *I* and $I_{p}$ become addicted at rates $\rho_{1}$ and $\rho_{2}$, respectively, then move into the compartment *A*(*t*). Thus, the total infected students population $N_{i}(t)$ is composed of


$$\begin{array}{c} N_{i}(t)=I(t)+I_{p}(t)+A(t). \end{array}$$ (2)

Commonly, during their break time or after class, students waste their study time sharing their opinions about ongoing trending videos on social media or debating about an actor’s role in particular episodes broadcast, for instance, on national TV, Showmax, Netflix, or others. The topics are usually brought out by infected students (the active/addicted subscribers), of which the non-subscribers (the susceptible) feel clueless about. As a result, the exposed susceptible develops a curiosity and search for the content in the social platform indicated. We thus assume that a susceptible student becomes infected through infected peer’s influence during contacts; and this occurs, at a rate η with an active subscriber *I*, or at a rate $\eta_{p}$ with $I_{p}$ subscriber, or with an addicted subscriber *A*, at a rate $\eta_{a}$. Note that $0<\eta_{p}<\eta <1<\eta_{a}$, to just classify the level of infectiousness of each infected individual. Moreover, infection may also occur through a direct contact with the contents $M_{1}(t),M_{2}(t),...,M_{n}(t)$, when a susceptible student independently of peer’s influences decides to create an account on these platforms; and this, at the rates $\eta_{k}$, 1≤k≤n, respectively. Usually, this spans from students who priorly were willing to join those entertaining channels but never had access to free Wi-Fi as that of the institution. The force of infection is thus given by


$$\begin{array}{c} \lambda =\underset{\text{peer-to-peer transmission}}{\underbrace{\frac{\eta_{p}I_{p}(t)+\eta I(t)+\eta_{a}A(t)}{N(t)}}}\;+\underset{\text{student-to-media transmission}}{\underbrace{\sum_{k=1}^{n}(1-\varepsilon_{r})\eta_{k}M_{k}(t).}} \end{array}$$ (3)

In Eq (3), the parameter $\varepsilon_{r}\in (0,1)$ accounts for the level of the regulation of social media accessibility within the institution, whereas $N(t)=N_{s}(t)+N_{i}(t)$ is the total student population.

We next assume that the infected students from *I*(*t*) and *A*(*t*) compartments contribute the most into the addictive contents’ growth by frequently sharing or uploading contents in each of the social media platform ${ℳ}_{k}$, at the rates θ and $\theta_{a}$, respectively. However, these contents $M_{1}(t),M_{2}(t),...,M_{n}(t)$ progressively lose students’ interest or attention, at a rate $\sigma_{k}$, 1≤k≤n, respectively. Students from $I_{p}(t)$, *I*(*t*), and *A*(*t*) are excluded from the institution due to the poor academic performance, and leave the institution, at the rates $\delta_{1}$, $\delta_{2}$, and $\delta_{3}$, respectively. Whilst students are recruited through purely susceptible compartment *S*(*t*), at a constant ra*t*e Λ, a non-social media rela*t*ed exit from the institution occurs in all compartments, at a rate μ.

Note that for “mathematical convenience”, we will opt to analyse the model, considering two most use social media within a given institution; that is, *n* = 2. The diagram in Fig 1 illustrates the “infection” dynamics within the total students population *N*(*t*)

**Fig 1:**
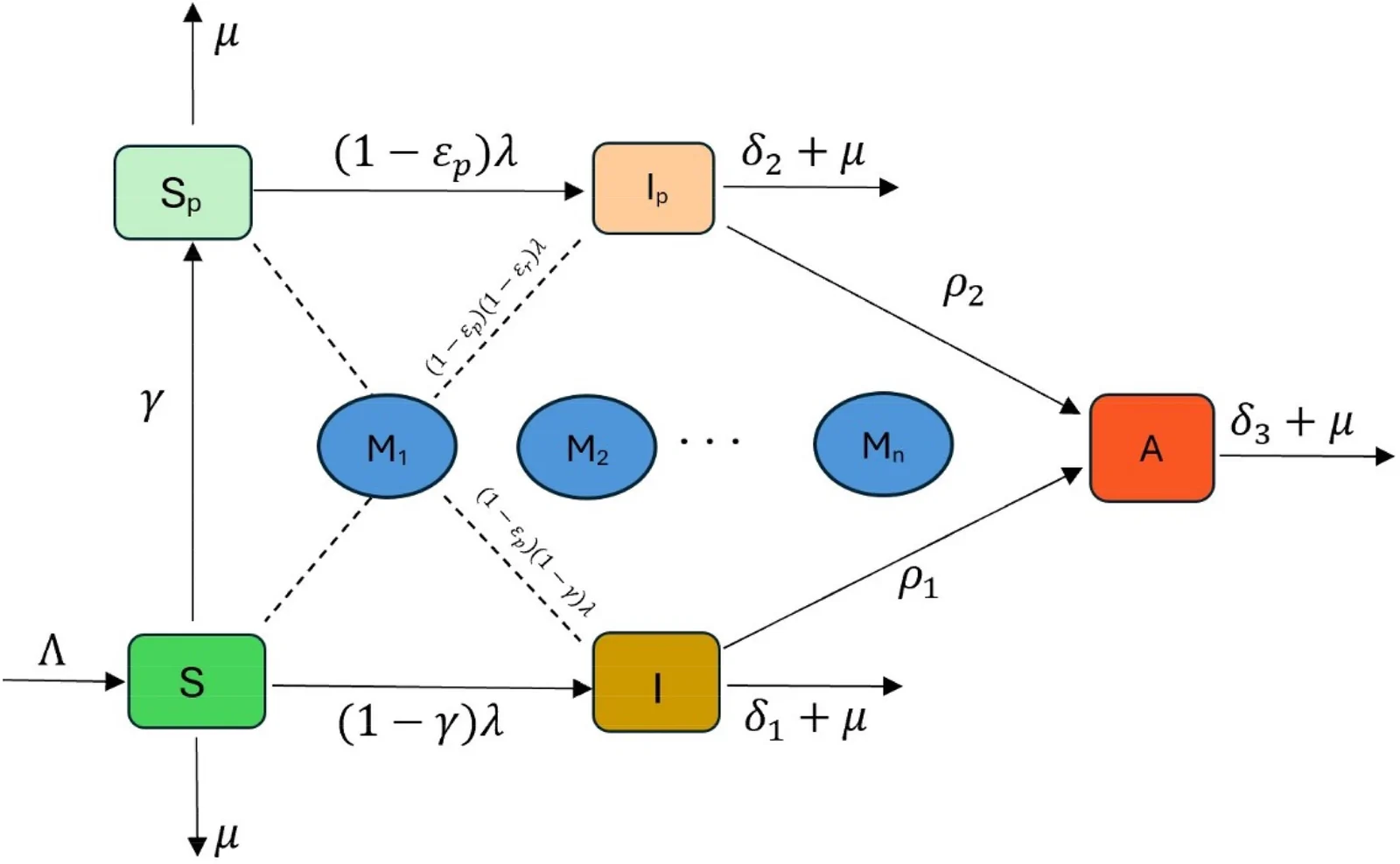
The model diagram illustrating the dynamics of social media addiction within the student population. We have omitted some transfers description to keep the diagram concise.

and the corresponding system of equations is given in Eq (4):


$$\begin{array}{c} \begin{array}{cc} \frac{dS(t)}{dt} & =\Lambda -\gamma S(t)-(1-\gamma )\lambda S(t)-\mu S(t)\\ \;\frac{dS_{p}(t)}{dt} & =\gamma S(t)-(1-\varepsilon_{p})\lambda S_{p}(t)-\mu S_{p}(t)\\ \;\frac{dI(t)}{dt} & =(1-\gamma )\lambda S(t)-(\rho_{1}+\mu +\delta_{1})I(t)\\ \;\frac{dI_{p}(t)}{dt} & =(1-\varepsilon_{p})\lambda S_{p}(t)-(\rho_{2}+\mu +\delta_{2})I_{p}(t)\\ \;\frac{dA(t)}{dt} & =\rho_{1}I(t)+\rho_{2}I_{p}(t)-(\mu +\delta_{3})A(t)\\ \;\frac{dM_{1}(t)}{dt} & =\theta I(t)+\theta_{a}A(t)-\sigma_{1}M_{1}(t)\\ \;\frac{dM_{2}(t)}{dt} & =\theta I(t)+\theta_{a}A(t)-\sigma_{2}M_{2}(t) \end{array}\} \end{array}$$ (4)

appended with the non-negative initial conditions: *S*(0)=*S*^0^ > 0, $S_{p}(0)=S_{p}^{0}>0$, $I_{p}(0)=I_{p}^{0}>0$, *I*(0)=*I*^0^ > 0, *A*(0)=*A*^0^ > 0, $M_{1}(0)=M_{1}^{0}>0,$ and $M_{2}(0)=M_{2}^{0}>0.$ The biological interpretation of the state variables and parameters appearing in system 4 are presented in Table 1.

**Table 1:** Description of the model state variables and parameters.

Variables	Description
$N_{i}(t)$	Total infected students
$N_{s}(t)$	Total susceptible students
*N*(*t*)	Total students population
*S*(*t*)	Total purely susceptible students
$S_{p}(t)$	Total Susceptible students under awareness programmes
$I_{p}(t)$	Total number of infected students due awareness failure
*I*(*t*)	Total number of infected students not enrolled in awareness programmes
*A*(*t*)	Total number of addicted students
$M_{k}(t)$	Total amount of addictive contents in social media ${ℳ}_{k}$
Parameters	Description
Λ	Recruitment rate
μ	Natural exit rate
η	Effective contact rate between *I* and *S* or $S_{p}$
$\eta_{p}$	Effective contact rate between $I_{p}$ and *S* or $S_{p}$
$\eta_{a}$	Effective contact rate between *A* and *S* or $S_{p}$
$\eta_{k}$	Effective contact rate between $M_{k}$ and *S* or $S_{p}$
$\varepsilon_{p}$	Awareness programmes efficacy level
$\varepsilon_{r}$	Social Media accessibility regulation rate
γ	Social Media use awareness programmes enrolment rate
θ	Contribution of addictive $M_{k}(t)$ growth by *I* student
$\theta_{a}$	Contribution of addictive $M_{k}(t)$ growth by *A* student
$\rho_{1},\rho_{2}$	Progression rate from *I*(*t*), $I_{p}$ state to *A*(*t*) state
$\delta_{1},\delta_{2},\delta_{3}$	Exclusion rate in $I(t),I_{p}(t),A(t)$ compartment due to ${ℳ}_{k}$ use
$\sigma_{1},\sigma_{2}$	Natural decay rate of interest of content $M_{1}(t),M_{2}(t)$

## Qualitative analysis of the model

The qualitative analysis of the model is structured in the following three subsections:

### Positivity of the model solutions

We claim that

**Theorem 0.1.** *All solutions of the model system (4) starting with non-negative initial conditions will remain non-negative for all time t > 0.*

*Proof.* We proceed considering the first equation of the system (4) as an inequality, that is


$$\begin{array}{c} \frac{dS(t)}{dt}\geq -(\gamma +(1-\gamma )\lambda +\mu )S(t). \end{array}$$ (5)

Now, solving the inequality (5) gives


$$\begin{array}{c} S(t)\geq S(0)\exp\left(-\int_{0}^{t}(\gamma +\mu +(1-\gamma )\lambda (u))du\right)\geq 0,\;\text{for}\;S(0)>0. \end{array}$$ (6)

Similarly, from the second equation of (4), we have


$$\begin{array}{c} \frac{dS_{p}(t)}{dt}=\gamma S(t)-(1-\varepsilon_{p})\lambda S_{p}(t)-\mu S_{p}(t)\geq -(\mu +(1-\varepsilon_{p})\lambda )S_{p}(t). \end{array}$$ (7)

Which yields


$$\begin{array}{c} S_{p}(t)\geq S_{p}(0)\exp\left(-\int_{0}^{t}(\mu +(1-\varepsilon_{p})\lambda (u))du\right)\geq 0,\;\text{for}\;S_{p}(0)>0. \end{array}$$ (8)

Following the same analogy, the remaining five equations of (4) can be re-written and their corresponding state variable can be proven non-negative, for any given non-negative initial condition. Thus, I(t)≥0, $I_{p}(t)\geq 0$, A(t)≥0, $M_{1}(t)\geq 0$, and $M_{2}(t)\geq 0$ also hold for all the time *t* > 0.

In sum, all solutions of the model are non-negative provided that their initial condition are defined non-negative.

### Boundedness of the model solutions

We claim that

**Theorem 0.2.** *All solutions of the model (4) are bounded in the dynamical meaningful region* $\Omega =\Omega_{s}\times \Omega_{k}$*, where* $\Omega_{s}\subseteq {\mathbb{R}}_{+}^{5}$*,* $\Omega_{k}\subseteq {\mathbb{R}}_{+}$*, such that* $\Omega_{k}=\left\{M_{k}\in {\mathbb{R}}_{+},0\leq M_{k}(t)\leq \frac{(\theta +\theta_{a})\Lambda }{\mu \sigma_{k}}\right\}$*, k = 1, 2 and* $\Omega_{s}=\left\{(S(t),S_{p}(t),I_{p}(t),I(t),A(t))\in {\mathbb{R}}_{+}^{5}:N(t)\leq \frac{\Lambda }{\mu }\right\}$*.*

*Proof.* We note that adding the first five equations of system (4) and solving for the resulting equation gives


$$\begin{array}{c} N(t)\leq \frac{\Lambda }{\mu }+\left(N(0)-\frac{\Lambda }{\mu }\right)e^{-\mu t}, \end{array}$$ (9)

which, whenever $N(0)\leq \frac{\Lambda }{\mu }$ or $N(0)\geq \frac{\Lambda }{\mu }$, yields $N(t)\leq \frac{\Lambda }{\mu }$, as t→∞.

Moreover, considering each of the last two equations of (4), we obtain the inequality


$$\begin{array}{c} M_{k}(t)\leq \frac{(\theta +\theta_{a})\Lambda }{\mu \sigma_{k}}+\left(M_{k}(0)-\frac{(\theta +\theta_{a})\Lambda }{\mu \sigma_{k}}\right)e^{-\sigma_{k}t},\;k=1,2 \end{array}$$ (10)

from which, we deduce that independently of the initial condition $M_{k}(0)>0$, the state variable $M_{k}(t)$ converges to $\frac{(\theta +\theta_{a})\Lambda }{\mu \sigma_{k}}$, as time *t* goes. □

In sum, all the solutions of the model (4) are positive and bounded in the dynamically meaningful region Ω. The region Ω is thus said to be positively invariant, dynamically meaningful, and attractive. We then carry out the model analyses in the region Ω.

### The Model equilibrium points and stability analysis

The model admits two typical equilibrium points: A social media-free equilibrium point (SMFE) and persistent addiction equilibrium (PAE) point. Their coordinates are determined and their stability conditions are rigorously analysed through the model key parameter, i.e., the control reproduction number ${ℛ}_{c}$.

#### The SMFE point and its stability analysis.

In the absence of excessive social media use, that is λ=0, the system (4) has a social media-free equilibrium (SMFE) point given by


$$\begin{array}{c} {𝒟}_{0}=\left(\frac{\Lambda }{\gamma +\mu },\frac{\gamma \Lambda }{\mu (\gamma +\mu )},0,0,0,0,0\right). \end{array}$$ (11)

It is easy to see that ${𝒟}_{0}$ is within the basin of the region Ω; i.e., ${𝒟}_{0}\in \Omega$.

#### The control reproduction number.

The control reproduction number ${ℛ}_{c}$ of the model is calculated using the Next-Generation Matrix (NGM) method in [15]. Thus, the NGM of the model is obtained from the transmission matrix *F* and the transition matrix *V*, which evaluated at ${𝒟}_{0}$ are given by


$$\begin{array}{c} F=\left(\begin{array}{ccccc} \eta f_{1} & \eta_{p}f_{1} & \eta_{a}f_{1} & \eta_{1}f_{1}f_{2} & \eta_{2}f_{1}f_{2}\\ \eta f_{3} & \eta_{p}f_{3} & \eta_{a}f_{3} & \eta_{1}f_{2}f_{3} & \eta_{2}f_{2}f_{3}\\ 0 & 0 & 0 & 0 & 0\\ 0 & 0 & 0 & 0 & 0\\ 0 & 0 & 0 & 0 & 0 \end{array}\right), \end{array}$$

where $f_{1}=\frac{(1-\gamma )\mu }{\gamma +\mu }$, $f_{2}=\frac{(1-\varepsilon_{r})\Lambda }{\mu }$ and $f_{3}=\frac{(1-\varepsilon_{a})\gamma }{\gamma +\mu }$, and


$$\begin{array}{ccc} V & = & \left(\begin{array}{ccccc} a & 0 & 0 & 0 & 0\\ 0 & b & 0 & 0 & 0\\ -\rho_{1} & -\rho_{2} & c & 0 & 0\\ -\theta & 0 & -\theta_{a} & \sigma_{1} & 0\\ -\theta & 0 & -\theta_{a} & 0 & \sigma_{2} \end{array}\right), \end{array}$$

where $a=\rho_{1}+\delta_{1}+\mu$, $b=\rho_{2}+\delta_{2}+\mu$, and $c=\delta_{3}+\mu$, so that


$$\begin{array}{c} V^{-1}=\left(\begin{array}{ccccc} \frac{1}{a} & 0 & 0 & 0 & 0\\ 0 & \frac{1}{b} & 0 & 0 & 0\\ \frac{\rho_{1}}{ac} & \frac{\rho_{2}}{bc} & \frac{1}{c} & 0 & 0\\ \frac{\rho_{1}\theta_{a}+\theta c}{ac\sigma_{1}} & \frac{\rho_{2}\theta_{a}}{bc\sigma_{1}} & \frac{\theta_{a}}{c\sigma_{1}} & \frac{1}{\sigma_{1}} & 0\\ \frac{\rho_{1}\theta_{a}+\theta c}{ac\sigma_{2}} & \frac{\rho_{2}\theta_{a}}{bc\sigma_{2}} & \frac{\theta_{a}}{c\sigma_{2}} & 0 & \frac{1}{\sigma_{2}} \end{array}\right). \end{array}$$

The next generation matrix is then given by


$$\begin{array}{c} FV^{-1}=\left(\begin{array}{ccccc} A_{11} & A_{12} & \frac{\eta_{a}f_{1}}{c}+\frac{\eta_{1}f_{1}f_{2}\theta_{a}}{c\sigma_{1}}+\frac{\eta_{2}f_{1}f_{2}\theta_{a}}{c\sigma_{2}} & \frac{\eta_{1}f_{1}f_{2}\theta_{a}}{\sigma_{1}} & \frac{\eta_{2}f_{1}f_{2}\theta_{a}}{\sigma_{2}}\\ A_{21} & A_{22} & \frac{\eta_{a}f_{3}}{c}+\frac{\eta_{1}f_{2}f_{3}\theta_{a}}{c\sigma_{1}}+\frac{\eta_{2}f_{2}f_{3}\theta_{a}}{c\sigma_{2}} & \frac{\eta_{1}f_{2}f_{3}\theta_{a}}{\sigma_{1}} & \frac{\eta_{2}f_{2}f_{3}\theta_{a}}{\sigma_{2}}\\ 0 & 0 & 0 & 0 & 0\\ 0 & 0 & 0 & 0 & 0\\ 0 & 0 & 0 & 0 & 0 \end{array}\right), \end{array}$$

whose characteristic polynomial is


$$\begin{array}{c} X^{2}-(A_{11}+A_{22})X+\underset{=0}{\underbrace{\left(A_{11}A_{22}-A_{21}A_{12}\right)}}=0, \end{array}$$ (12)

where


$$\begin{array}{cc} A_{11} & =\frac{\eta f_{1}}{a}+\frac{\eta_{a}f_{1}\rho_{1}}{ac}+\frac{\eta_{1}f_{1}f_{2}(\rho_{1}\theta_{a}+\theta c)}{ac\sigma_{1}}+\frac{\eta_{2}f_{1}f_{2}(\rho_{1}\theta_{a}+\theta c)}{ac\sigma_{2}}\\ A_{12} & =\frac{\eta_{p}f_{1}}{b}+\frac{\eta_{a}f_{1}\rho_{2}}{bc}+\frac{\eta_{1}f_{1}f_{2}\rho_{2}\theta_{a}}{bc\sigma_{1}}+\frac{\eta_{2}f_{1}f_{2}\rho_{2}\theta_{a}}{bc\sigma_{2}}\\ A_{21} & =\frac{\eta f_{3}}{a}+\frac{\eta_{a}f_{3}\rho_{1}}{ac}+\frac{\eta_{1}f_{2}f_{3}(\rho_{1}\theta_{a}+\theta c)}{ac\sigma_{1}}+\frac{\eta_{2}f_{1}f_{2}(\rho_{1}\theta_{a}+\theta c)}{ac\sigma_{2}}\\ A_{22} & =\frac{\eta_{p}f_{3}}{b}+\frac{\eta_{a}f_{3}\rho_{2}}{bc}+\frac{\eta_{1}f_{2}f_{3}\rho_{2}\theta_{a}}{bc\sigma_{2}}+\frac{\eta_{2}f_{1}f_{2}\rho_{2}\theta_{a}}{bc\sigma_{2}}. \end{array}$$

Thus, the control reproduction number ${ℛ}_{c}$ of our model defined as the spectral radius $\rho (FV^{-1})$ of the next generation matrix *F V*^–1^ is given by


$$\begin{array}{c} {ℛ}_{c}=\underset{peers^{\prime }\;influence\;contacts\;contribution:\;{ℛ}_{p}}{\underbrace{\frac{\eta f_{1}}{a}+\frac{\eta_{p}f_{3}}{b}+\frac{\eta_{a}f_{1}\rho_{1}}{ac}+\frac{\eta_{a}f_{3}\rho_{2}}{bc}}}+\underset{social\;media\;contacts\;contribution:\;{ℛ}_{m}}{\underbrace{\sum_{k=1}^{2}\frac{f_{1}f_{2}\eta_{k}(c\theta +\theta_{a}\rho_{1})}{ac\sigma_{k}}+\sum_{k=1}^{2}\frac{f_{2}f_{3}\eta_{k}\theta_{a}\rho_{2}}{bc\sigma_{k}}}}. \end{array}$$ (13)

The control reproduction number (${ℛ}_{c}$) represents the expected number of new students who would develop excessive social media use habits due to the influence of a single affected student, in a context where awareness and regulation interventions are implemented. If ${ℛ}_{c}>1$, it implies that each distracted student can, on average, influences more than one other student to adopt similar behaviours, resulting in a sustained spread of distraction and decline in academic performance. Conversely, ${ℛ}_{c}<1$ indicates that the spread of such behaviours diminishes over time, with students gradually returning to healthier study patterns. From a policy perspective, this means that awareness programs (γ, $\varepsilon_{p}$) and regulatory measures ($\varepsilon_{r}$) must be strengthened to reduce ${ℛ}_{c}$ below unity, thereby preventing the persistence of academic distraction.

We next claim that

**Theorem 0.3.** *The SMFE point* ${𝒟}_{0}$ *of the system (4) is locally asymptotically stable whenever* ${ℛ}_{c}\leq 1$ *and unstable otherwise.*

*Proof.* Following the techniques of authors in [15], we restrict the local stability of the SMFE point ${𝒟}_{0}$ to the stability of the matrix F−V given by


$$\begin{array}{c} F-V=\left(\begin{array}{ccccc} \eta f_{1}-a & \eta_{p}f_{1} & \eta_{a}f_{1} & \eta_{1}f_{1}f_{2} & \eta_{2}f_{1}f_{2}\\ \eta f_{3} & \eta_{p}f_{3}-b & \eta_{a}f_{3} & \eta_{1}f_{2}f_{3} & \eta_{2}f_{2}f_{3}\\ \rho_{1} & \rho_{2} & -c & 0 & 0\\ \theta & 0 & \theta_{a} & -\sigma_{1} & 0\\ \theta & 0 & \theta_{a} & 0 & -\sigma_{2} \end{array}\right), \end{array}$$

whose eigenvalues are the roots of its characteristic polynomial


$$\begin{array}{c} \lambda^{5}+a_{4}\lambda^{4}+a_{3}\lambda^{3}+a_{2}\lambda^{2}+a_{1}\lambda +a_{0}=0, \end{array}$$ (14)

where


$$\begin{array}{cc} a_{4} & =a\left(1-\frac{\eta f_{1}}{a}\right)+b\left(1-\frac{\eta_{p}f_{3}}{b}\right)+c+\sigma_{1}+\sigma_{2}\\ a_{3} & =ab\left(1-\frac{\eta f_{1}}{a}-\frac{\eta_{p}f_{3}}{b}\right)+ac\left(1-\frac{\eta_{a}f_{1}\rho_{1}}{a}-\frac{\eta f_{1}}{a}\right)+a\sum_{k=1}^{2}\sigma_{k}\left(1-\frac{\eta_{k}f_{1}f_{2}\theta }{a\sigma_{k}}-\frac{\eta f_{1}}{a}\right)\\ & +bc\left(1-\frac{\eta_{p}f_{3}\rho_{2}}{b}-\frac{\eta_{p}f_{3}}{b}\right)+b\sum_{k=1}^{2}\sigma_{k}\left(1-\frac{\eta_{p}f_{3}}{b}\right)+c\sigma_{1}\sigma_{2}\left(\frac{1}{c}+\frac{1}{\sigma_{1}}+\frac{1}{\sigma_{2}}\right)\\ a_{2} & =abc\left(1-\frac{\eta f_{1}}{a}+\frac{\eta f_{3}}{b}+\frac{\eta_{a}\rho_{1}f_{1}}{ac}+\frac{\eta_{a}\rho_{2}f_{3}}{bc}\right)+ab\sum_{k=1}^{2}\sigma_{k}\left(1-\frac{f_{1}f_{2}\eta_{k}\theta }{a\sigma_{k}}-\frac{\eta f_{1}}{a}-\frac{\eta_{p}f_{3}}{b}\right)\\ & +\;ac\sum_{k=1}^{2}\sigma_{k}\left(1-\frac{f_{1}f_{2}\eta_{k}\theta_{3}\rho_{1}}{ac\sigma_{k}}-\frac{\eta f_{1}}{a}-\frac{\eta_{a}f_{1}\rho_{1}}{ac}\right)+bc\sum_{k=1}^{2}\sigma_{k}\left(1-\frac{f_{2}f_{3}\eta_{k}\theta_{3}\rho_{2}}{bc\sigma_{k}}-\frac{\eta_{p}f_{3}}{b}-\frac{\eta_{a}f_{3}\rho_{2}}{bc\sigma_{k}}\right)\\ & +\;a\sigma_{1}\sigma_{2}\left(1-\frac{f_{1}f_{2}\eta_{1}\theta }{a\sigma_{1}}-\frac{\eta f_{1}}{a}\right)+\frac{\sigma_{1}\sigma_{2}}{ac}+\frac{\sigma_{1}\sigma_{2}}{ab}\\ a_{1} & =abc\sum_{k=1}^{2}\sigma_{k}\left(1-\frac{\eta f_{1}}{a}-\frac{\eta_{p}f_{3}}{b}-\frac{\eta_{a}f_{1}\rho_{1}}{ac}-\frac{\eta_{a}f_{3}\rho_{2}}{bc}-\frac{f_{1}f_{2}\eta_{k}\theta }{a\sigma_{k}}-\sum_{j=1}^{2}\frac{f_{1}f_{2}\eta_{k}\theta_{3}\rho_{j}}{ac\sigma_{k}}\right)\\ & +ab\sigma_{1}\sigma_{2}\left(1-\frac{\eta f_{1}}{a}-\frac{\eta_{p}f_{3}}{b}-\sum_{j=1}^{2}\frac{f_{1}f_{2}\eta_{2}\theta }{a\sigma_{j}}\right)+bc\sigma_{1}\sigma_{2}\left(1-\frac{\eta_{p}f_{3}}{b}-\frac{\eta_{a}f_{3}\rho_{2}}{ac}-\sum_{k=1}^{2}\frac{f_{2}f_{3}\eta_{k}\theta_{3}\rho_{2}}{bc\sigma_{k}}\right)\\ & +ac\sigma_{1}\sigma_{2}\left(1-\frac{\eta f_{1}}{a}-\frac{\eta_{a}f_{1}\rho_{1}}{ac}-\sum_{k=1}^{2}\frac{f_{1}f_{2}\eta_{k}\theta }{a\sigma_{k}}-\sum_{k=1}^{2}\frac{f_{1}f_{2}\eta_{k}\theta_{3}\rho_{1}}{ac\sigma_{k}}\right)\\ a_{0} & =abc\sigma_{1}\sigma_{2}(1-{ℛ}_{c}). \end{array}$$

We note that the coefficient *a*_0_ as well as *a*_1_, *a*_2_, *a*_3_, and *a*_4_, are all non-negative, provided that ${ℛ}_{c}<1$. Thus, all the roots of polynomial (14) are negative or have negative real part, whenever ${ℛ}_{c}<1$. The matrix F−V is then stable. The SMFE point ${𝒟}_{0}$ is therefore locally asymptotically stable, whenever ${ℛ}_{c}<1$ and unstable otherwise.

Theorem 0.3 established that the social media harmful impact on students academic performance can be eradicated in time should ${ℛ}_{c}$ be maintained under unit; and this if the initial size of each infected state variable stays in the basin of the dynamically meaningful region Ω. To ensure that such eradication is independent of the initial sizes of the infected student population that are registered into the susceptible students population, the global stability of the SMFE point needs to be guaranteed. We investigate that as follows:□

We use the Comparison Theorem in [16], by letting $𝒳=[I(t),I_{p}(t),A(t),M_{1}(t),M_{2}(t){]}^{⊤}$ be a vector of the infected compartments of the model (4) so that


$$\begin{array}{c} \frac{d𝒳}{dt}=(F-V){𝒳}^{⊤}-Z{𝒳}^{⊤}, \end{array}$$ (15)

where


$$\begin{array}{c} Z=\left(\begin{array}{c} d_{1}\left(\frac{\mu }{\gamma +\mu }-\frac{S(t)}{N(t)}\right)+d_{2}\left(\frac{\Lambda }{\gamma +\mu }-S(t)\right)\\ d_{3}\left(\frac{\gamma }{\gamma +\mu }-\frac{S_{p}(t)}{N(t)}\right)+d_{4}\left(\frac{\gamma \Lambda }{(\gamma +\mu )\mu }-S_{p}(t)\right)\\ 0\\ 0\\ 0 \end{array}\right). \end{array}$$

so that $d_{1}=(\eta I+\eta_{p}I_{p}+\eta_{a}A)(1-\gamma )$,   $d_{2}=(1-\gamma )(1-\varepsilon_{r})(\eta_{1}M_{1}(t)+\eta_{2}M_{2}(t))$,  $d_{3}=(\eta I+\eta_{p}I_{p}+\eta_{a}A)(1-\varepsilon_{a})$, and $d_{4}=(\eta I+\eta_{p}I_{p}+\eta_{a}A)(1-\varepsilon_{a})(1-\varepsilon_{r}).$

One notes that *d*_1_, *d*_2_, *d*_3_, and *d*_4_ are all positive as well as the other factors since $\frac{\mu }{\gamma +\mu }\geq \frac{S(t)}{N(t)}$, $\frac{\Lambda }{\gamma +\mu }\geq S(t)$, $\frac{\gamma \Lambda }{(\gamma +\mu )\mu }\geq S_{p}(t)$ and $\frac{\gamma }{\gamma +\mu }\geq \frac{S_{p}(t)}{N(t)}$, for all time *t* > 0.

Thus, the matrix *Z* is a non-negative matrix. Therefore, Eq (15) yields the inequality


$$\begin{array}{c} \frac{d}{dt}\left(\begin{array}{c} I(t)\\ I_{p}(t)\\ A(t)\\ M_{1}(t)\\ M_{2}(t) \end{array}\right)\leq (F-V)\left(\begin{array}{c} I(t)\\ I_{p}(t)\\ A(t)\\ M_{1}(t)\\ M_{2}(t) \end{array}\right). \end{array}$$ (16)

Since Theorem 0.3 guarantees that all the eigenvalues of the matrix F−V have negative real part, whenever ${ℛ}_{c}\leq 1$, it then follows that the linearised differential inequality system (16) is stable. Hence, by the Comparison Theorem in [16], we have


$$\begin{array}{c} \lim_{t⟶\infty }(I(t),I_{p}(t),A(t),M_{1}(t),M_{2}(t))=(0,0,0,0,0). \end{array}$$ (17)

Now, substituting the above result into (4) implies that *S*(*t*) and $S_{p}(t)$ approach their upper bounds $\frac{\Lambda }{\gamma +\mu }$ and $\frac{\gamma \Lambda }{(\gamma +\mu )\mu }$, respectively, as time *t* goes to infinity. Thus,


$$\begin{array}{c} \lim_{t⟶\infty }(S(t),S_{p}(t),I(t),I_{p}(t),A(t),M_{1}(t),M_{2}(t))=\left(\frac{\Lambda }{\gamma +\mu },\frac{\gamma \Lambda }{\mu (\gamma +\mu )},0,0,0,0,0\right)={𝒟}_{0}. \end{array}$$ (18)

We therefore claim that

**Theorem 0.4.** *The SMFE point* ${𝒟}_{0}$ *of the model (4) is globally asymptotically stable whenever* ${ℛ}_{c}\leq 1.$

Results from Theorem 0.3 and Theorem 0.4 indicate that if control interventions implemented (either single or in combined form) are strong enough to keep ${ℛ}_{c}$ below one, then harmful patterns of social media use will naturally decline, allowing students to regain focus on academic work. Therefore, they would produce a desired academic performance. This might demand a comprehensive and effective social media control policies within the higher learning institution.

#### The persistent addiction equilibrium point and its stability analysis.

The PAE point ${ℰ}^{*}$ corresponds to a situation where the excessive use of social media persists within the student population alongside no variation in the size of each sub-population with respect to time *t* > 0. The coordinates of ${ℰ}^{*}$ are thus obtained by equa*t*ing the right-hand side of the system (4) to zero and then solving for $S^{*},S_{p}^{*},I^{*},I_{p}^{*},A^{*},M_{1}^{*}$, and $M_{2}^{*}$. This gives


$$\begin{array}{c} {ℰ}^{*}=(S^{*},S_{p}^{*},I^{*},I_{p}^{*},A^{*},M_{1}^{*},M_{2}^{*}), \end{array}$$ (19)

where


$$\begin{array}{cc} S^{*} & =\frac{\mu \Lambda }{\left(\gamma +\mu \right)}.\frac{1}{f_{1}\lambda^{*}+\mu },\;S_{p}^{*}=\frac{\mu \gamma^{2}\Lambda }{(\gamma +\mu {)}^{2}}.\frac{1}{\left[\left(\frac{\mu \gamma }{\gamma +\mu }+f_{3}\lambda^{*}\right)(f_{1}\lambda^{*}+\mu )\right]},\\ I^{*} & =\frac{f_{1}\Lambda }{a}.\frac{\lambda^{*}}{f_{1}\lambda^{*}+\mu },\;\;\;I_{p}^{*}=\frac{f_{3}\mu \gamma \Lambda }{b(\gamma +\mu )}.\frac{\lambda^{*}}{\left[\left(\frac{\mu \gamma }{\gamma +\mu }+f_{3}\lambda^{*}\right)(f_{1}\lambda^{*}+\mu )\right]},\\ A^{*} & =\frac{f_{1}\rho_{1}\Lambda }{ac}.\frac{\lambda^{*}}{f_{1}\lambda^{*}+\mu }+\frac{\mu f_{3}\rho_{2}\gamma \Lambda }{bc(\gamma +\mu )}.\frac{\lambda^{*}}{\left[\left(\frac{\mu \gamma }{\gamma +\mu }+f_{3}\lambda^{*}\right)(f_{1}\lambda^{*}+\mu )\right]},\\ M_{1}^{*} & =\frac{f_{1}\Lambda (c\theta +\rho_{1}\theta_{a})}{ac\sigma_{1}}.\frac{\lambda^{*}}{f_{1}\lambda^{*}+\mu }+\frac{\mu f_{3}\rho_{2}\theta_{a}\gamma \Lambda }{bc\sigma_{1}(\gamma +\mu )}.\frac{\lambda^{*}}{\left[\left(\frac{\mu \gamma }{\gamma +\mu }+f_{3}\lambda^{*}\right)(f_{1}\lambda^{*}+\mu )\right]},\\ M_{2}^{*} & =\frac{f_{1}\Lambda (c\theta +\rho_{1}\theta_{a})}{ac\sigma_{2}}.\frac{\lambda^{*}}{f_{1}\lambda^{*}+\mu }+\frac{\mu f_{3}\rho_{2}\theta_{a}\gamma \Lambda }{bc\sigma_{2}(\gamma +\mu )}.\frac{\lambda^{*}}{\left[\left(\frac{\mu \gamma }{\gamma +\mu }+f_{3}\lambda^{*}\right)(f_{1}\lambda^{*}+\mu )\right]}. \end{array}$$

These coordinates exist and are dynamically meaningful, if the endemic force of infection $\lambda^{*}$ remains positive. We argue that since the positivity of all solutions is guaranteed, so are the model equilibrium solutions. Thus, it follows that $\lambda^{*}>0$. This leads to the inequalities


$$\begin{array}{c} \frac{\lambda^{*}}{f_{1}\lambda^{*}+\mu }\leq \frac{\lambda^{*}}{\mu }\;\text{and}\;\frac{\lambda^{*}}{\left(f_{4}+f_{3}\lambda^{*}\right)(f_{1}\lambda^{*}+\mu )}\leq \frac{\lambda^{*}}{\mu f_{4}},\;\text{where}\;f_{4}=\frac{\mu \gamma }{\gamma +\mu }. \end{array}$$

Using both above inequalities, we manipulate the right-hand side of the coordinates of ${ℰ}^{*}$ to obtain the inequality $\lambda^{*}\phi (\lambda^{*})\leq {ℛ}_{c}\lambda^{*}$, that is, $\phi (\lambda^{*})\leq {ℛ}_{c}$. ϕ is a positive, continuous and decreasing function that maps on Ω to ${\mathbb{R}}_{+}$ (see Appendix).

Moreover, substituting all the coordinates of ${ℰ}^{*}$ into the right-hand side of


$$\begin{array}{c} \lambda^{*}=\frac{\eta I^{*}+\eta_{p}I_{a}^{*}+\eta_{a}A^{*}}{N^{*}}+(1-\varepsilon_{r})\eta_{1}M_{1}^{*}+(1-\varepsilon_{r})\eta_{2}M_{2}^{*}. \end{array}$$ (20)

and simplifying the resulting equation, gives $\lambda^{*}=\lambda^{*}\phi (\lambda^{*})$. Which yields two equalities: $\lambda_{1}^{*}=0$ or $1-\phi (\lambda_{2}^{*})=0$, where $\lambda_{2}^{*}\neq 0$. While the first solution corresponds to the social media-free equilibrium point ${𝒟}_{0}$, the second equation’s solutions are analysed through the Intermediate Value Theorem (I.V.T) as follows:

Let $𝒵(\lambda^{*})=1-\phi (\lambda^{*})$ be a continuously differentiable and positive definite function on $\lambda^{*}\in (0,\infty )$. From the precedent result $0<\phi (\lambda^{*})\leq {ℛ}_{c}$, it follows that 𝒵 is bounded,i.e., $1-{ℛ}_{c}\leq 𝒵(\lambda^{*})<1,$ for all $\lambda^{*}\in [0,\infty )$.

One notes that 𝒵 is an increasing function as ${𝒵}^{\prime }(\lambda^{*})=-\phi^{\prime }(\lambda^{*})>0$, since $\phi^{\prime }(\lambda^{*})<0$, for all $\lambda^{*}\in (0,\infty )$. In addition, $𝒵(0)=1-{ℛ}_{c}$ and $\lim_{\lambda^{*}⟶\infty }𝒵(\lambda^{*})=1$. It then follows from the I.V.T that, if ${ℛ}_{c}\leq 1$ then the equation $1-\phi (\lambda^{*})=0$ has no solution, whereas if ${ℛ}_{c}>1$ then $1-\phi (\lambda^{*})=0$ has at least one positive solution, corresponding to the PAE point. These analyses are graphically illustrated in Fig 2.

**Fig 2:**
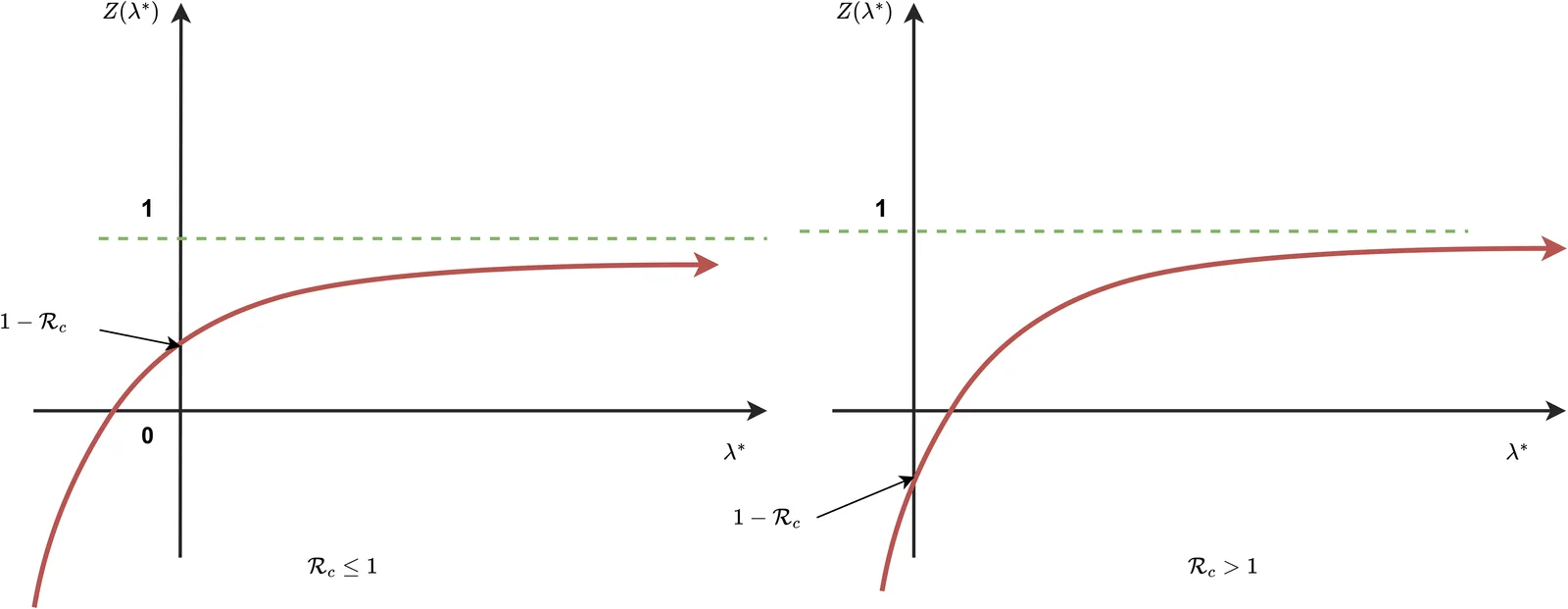
Simulations of the function 𝒵 illustrating the existence of the persistent addiction equilibrium point, when ${ℛ}_{c}>1$ and ${ℛ}_{c}\leq 1$.

We therefore claim that

**Lemma 0.5.** *The PAE point* ${ℰ}^{*}$ *of the model 4 exists and remains in the basin of the region* Ω*, whenever* ${ℛ}_{c}>1.$

We investigate the uniqueness and the local stability of ${ℰ}^{*}$ through Theorem 0.6. In case of more than one PAE point, a phenomenon known as a backward bifurcation will emerge at ${ℛ}_{c}=1$. In that situation, a stable PAE point co-exists with a stable SMFE point [17,18]. A direct implication of such scenario is that when a backward bifurcation occurs, achieving ${ℛ}_{c}<1$ may not be sufficient for eradicating the harmful social media use, if the number of the already-distracted students remains high. A condition which would mandate the necessity of combining control interventions, such as raising awareness enrolment rates and enforcing stricter accessibility controls, to ensure effective outcomes. However, in case of a unique PAE point the model will exhibits a forward bifurcation/transcritical bifurcation.

We hesitantly claim that

**Theorem 0.6.** *The PAE point* ${ℰ}^{*}$ *of the model is unique and locally asymptotically stable whenever* ${ℛ}_{c}>1.$

*Proof.* We apply the center manifold theory in [19] to the model, to prove the above claim. The model (4) is re-written as $\frac{dy}{dt}=G(y)$, where $G(y)=(g_{1},g_{2},g_{3},g_{4},g_{5},g_{6},g_{7})$. Hence, it follows that


$$\begin{array}{c} \begin{array}{cc} \frac{dy_{1}(t)}{dt} & =g_{1}=\Lambda -\gamma y_{1}(t)-(1-\gamma )\lambda y_{1}(t)-\mu y_{1}(t),\\ \;\frac{dy_{2}(t)}{dt} & =g_{2}=\gamma y_{1}(t)-(1-\varepsilon_{p})\lambda y_{2}(t)-\mu y_{2}(t),\\ \;\frac{dy_{3}(t)}{dt} & =g_{3}=(1-\gamma )\lambda y_{1}(t)-(\rho_{1}+\mu +\delta_{1})y_{3}(t),\\ \;\frac{dy_{4}(t)}{dt} & =g_{4}=(1-\varepsilon_{a})\lambda y_{2}(t)-(\rho_{2}+\mu +\delta_{2})y_{4}(t),\\ \;\frac{dz_{5}(t)}{dt} & =g_{5}=\rho_{1}y_{3}(t)+\rho_{2}y_{4}(t)-(\mu +\delta_{3})y_{5}(t),\\ \;\frac{dy_{6}(t)}{dt} & =g_{6}=\theta y_{3}(t)+\theta_{a}y_{5}(t)-\sigma_{1}y_{6}(t),\\ \;\frac{dy_{7}(t)}{dt} & =g_{7}=\theta y_{3}(t)+\theta_{a}y_{5}(t)-\sigma_{2}y_{7}(t), \end{array}\} \end{array}$$ (21)

Now, considering $c=\delta_{3}+\mu$ as the bifurcation parameter when ${ℛ}_{c}=1$, the Jacobian matrix ℬ of the system (21) evaluated at the SMFE point is given by


$$\begin{array}{c} ℬ=\left(\begin{array}{ccccccc} -(\gamma +\mu ) & 0 & -\eta f_{1} & -\eta_{p}f_{1} & -\eta_{a}f_{1} & -\eta_{1}f_{1}f_{2} & -\eta_{2}f_{1}f_{2}\\ \gamma & -\mu & -\eta f_{3} & -\eta_{p}f_{3} & -\eta_{a}f_{3} & -\eta_{1}f_{2}f_{3} & -\eta_{2}f_{2}f_{3}\\ 0 & 0 & \eta f_{1}-a & \eta_{p}f_{1} & \eta_{a}f_{1} & \eta_{1}f_{1}f_{2} & \eta_{2}f_{1}f_{2}\\ 0 & 0 & \eta f_{3} & \eta_{p}f_{3}-b & \eta_{a}f_{3} & \eta_{1}f_{2}f_{3} & \eta_{2}f_{2}f_{3}\\ 0 & 0 & \rho_{1} & \rho_{2} & -c & 0 & 0\\ 0 & 0 & \theta & 0 & \theta_{a} & -\sigma_{1} & 0\\ 0 & 0 & \theta & 0 & \theta_{a} & 0 & -\sigma_{2} \end{array}\right). \end{array}$$ (22)

Expanding det(ℬ−ζI) with respect to its second and first columns, respectively, gives the eigenvalues $\zeta_{1}=-\mu$ and $\zeta_{2}=-(\gamma +\mu )$, whereas the matrix ℬ is reduced to ${ℬ}_{5}$; which corresponds exactly to the aforementioned matrix F−V. This later has a simple eigenvalue zero, $\zeta_{3}=0$, when ${ℛ}_{c}=1$. The remaining eigenvalues are therefore the roots of the polynomial in Eq (14). Which are priorly proven all negative. Thus, the Jacobian matrix ℬ has a simple eigenvalue $\zeta_{3}=0$ and all other eigenvalues are negative. The centre manifold conditions are thus satisfied.

Let $𝒲=[{\bar{w}}_{1},{\bar{w}}_{2},...,{\bar{w}}_{7}{]}^{⊤}$ and $ℒ=[{\bar{l}}_{1},{\bar{l}}_{2},...,{\bar{l}}_{7}{]}^{⊤}$ be the right and the left eigenvectors corresponding to $\zeta_{3}=0$ so that


$$\begin{array}{c} 𝒲=\left(\begin{array}{c} -\frac{a}{\gamma +\mu }\\ -\frac{a}{\mu }\left(\frac{\gamma }{\gamma +\mu }+\frac{f_{3}}{f_{1}}\right)\\ 1\\ \frac{af_{3}}{bf_{1}}\\ \left(\frac{\rho_{1}}{c^{*}}+\frac{\rho_{2}af_{3}}{bc^{*}f_{1}}\right)\\ \frac{\left(\theta c^{*}+\theta_{a}\rho_{1}\right)}{\sigma_{1}c^{*}}+\frac{a\rho_{2}f_{3}\theta_{a}}{bc^{*}f_{1}\sigma_{1}}\\ \frac{\left(\theta c^{*}+\theta_{a}\rho_{1}\right)}{\sigma_{2}c^{*}}+\frac{a\rho_{2}f_{3}\theta_{a}}{bc^{*}f_{1}\sigma_{2}} \end{array}\right){\bar{w}}_{3},\;ℒ=\left(\begin{array}{c} 0\\ 0\\ 1\\ \frac{\rho_{2}a{ℛ}_{b}}{\rho_{1}b{ℛ}_{a}}\\ -\eta_{p}\left[1+\frac{\rho_{2}f_{3}a{ℛ}_{b}}{\rho_{1}f_{1}b{ℛ}_{a}}\right]\\ \frac{\eta_{1}f_{2}}{\sigma_{1}f_{1}}\left[1+\frac{\rho_{2}f_{3}a{ℛ}_{b}}{\rho_{1}f_{1}b{ℛ}_{a}}\right]\\ \frac{\eta_{2}f_{2}}{\sigma_{2}f_{1}}\left[1+\frac{\rho_{2}f_{3}a{ℛ}_{b}}{\rho_{1}f_{1}b{ℛ}_{a}}\right] \end{array}\right){\bar{l}}_{3} \end{array}$$ (23)

where ${\bar{w}}_{3}=1$ and ${\bar{l}}_{3}=1$.

Moreover, it can be verified that 𝒲·ℒ=1. The bifurcation coefficients a and b of the current model, following formula in [19], are given by


$$\begin{array}{cc} a & =\sum_{i,j,k=1}^{7}{\bar{l}}_{k}{\bar{w}}_{i}{\bar{w}}_{j}\frac{\partial^{2}g_{k}({𝒟}_{0})}{\partial y_{i}\partial y_{j}}=-\frac{af_{3}{\bar{l}}_{3}}{\Lambda {ℛ}_{a}}(\eta {\bar{w}}_{3}+\eta_{p}{\bar{w}}_{4}+\eta_{a}{\bar{w}}_{5})-\frac{af_{1}f_{2}{\bar{l}}_{3}}{\Lambda }(\eta_{1}{\bar{w}}_{6}+\eta_{2}{\bar{w}}_{7})\\ & -\frac{2f_{1}\mu {\bar{l}}_{3}}{\Lambda }(\eta {\bar{w}}_{3}^{2}+\eta_{p}{\bar{w}}_{4}^{2}+\eta_{a}{\bar{w}}_{5}^{2})-\left(1+\frac{f_{3}}{f_{1}}\frac{\gamma +\mu }{\gamma }\right)\frac{af_{2}f_{3}\mu {\bar{l}}_{4}}{\Lambda }(\eta_{1}{\bar{w}}_{6}+\eta_{2}{\bar{w}}_{7})\\ & -\frac{2f_{3}\mu {\bar{l}}_{4}}{\Lambda }(\eta {\bar{w}}_{3}^{2}+\eta_{p}{\bar{w}}_{4}^{2}+\eta_{a}{\bar{w}}_{5}^{2})<0,\\ b & =\sum_{i,k=1}^{7}{\bar{l}}_{k}{\bar{w}}_{i}\frac{\partial^{2}g_{k}({𝒟}_{0})}{\partial y_{i}\partial c^{*}}={\bar{l}}_{5}{\bar{w}}_{5}\frac{\partial^{2}g_{5}({𝒟}_{0})}{\partial y_{5}\partial c^{*}}=\eta_{p}\left(\frac{\rho_{1}}{c^{*}}+\frac{\rho_{2}af_{3}}{c^{*}bf_{1}}\right)\left[1+\frac{\rho_{2}f_{3}a{ℛ}_{b}}{\rho_{1}f_{1}b{ℛ}_{a}}\right]>0. \end{array}$$

Since a<0 and b>0, it follows from Theorem 4.1 in [19] that the model undergoes a forward transcritical bifurcation at ${ℛ}_{c}=1$; an exchange of stability between unstable SMFE point ${𝒟}_{0}$ and a stable PAE point ${ℰ}^{*}$ occurs. The model therefore has a unique PAE point. Which suggests that reducing ${ℛ}_{c}$ below one would be a sufficient condition for “detoxifying” the student population. This result is presented in Fig 3. □

**Fig 3:**
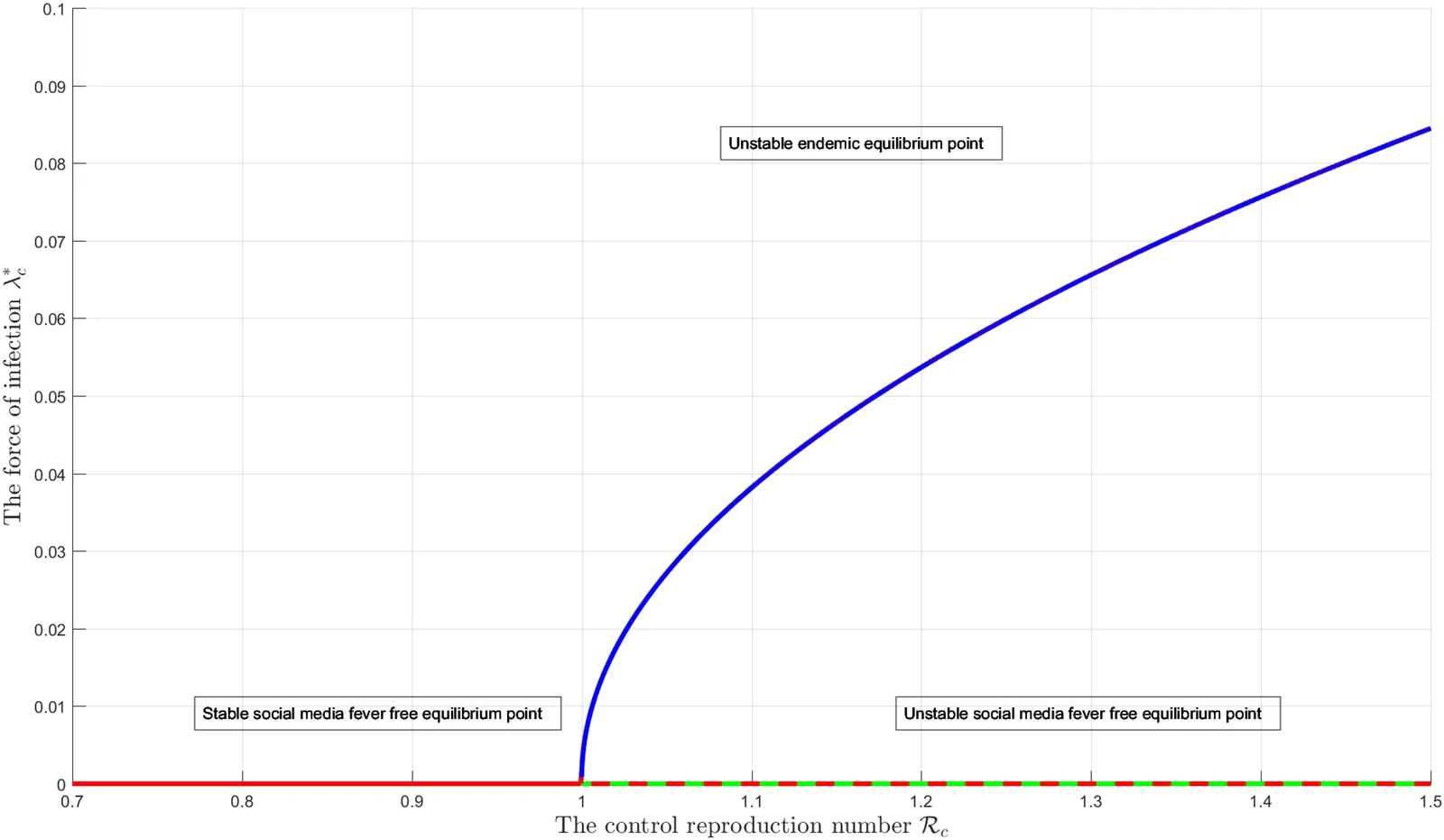
A bifurcation diagram illustrating a forward transcritical bifurcation of the endemic force infection $\lambda^{*}$ against the control reproduction number ${ℛ}_{c}$.

## Numerical analysis

To validate the qualitative results, we undertake numerical simulations of the model using some parameter values and initial total student population of *N*(0)=500, whom are homogeneously composed in majority of freshmen, repeated students, and second year students, all aged 18 years and above. While the first are completely naive and not aware that an obsession to the existing entertaining contents can infringe their well-being and their study time flows, a proportion of the second and the third group are identified as infected $\left(I(0),I_{p}(0)\right)$ and addicted (*A*(0)) subscribers. A student is identified as an infected if the cumulative time $t_{m}=\sum_{k=1}^{n}t_{k}$ spent on social media platforms ranges between 1–3 hours per day, and an addicted if $t_{m}$ is greater than 3 hours. The study is then restricted to the students enrolled in mathematics and math-education curriculum at the University of the Free State in year 2025 [20], and the initial addiction/high-use compartments have been initialized using empirical prevalence ranges (5%−−20%) reported in recent university surveys [6,21,22]. On the other hand, we assume that each student is initially exposed to $M_{1}(0)=10^{5}$ and $M_{2}(0)=10^{3}$ addictive contents per day, in both most used social platforms, respectively.

Given the exploratory nature of this study and due to the scarcity of empirical estimates in the literature, several model parameters and certain initial condition values are necessarily assumed to preserve behavioural consistency and mathematical feasibility. The adopted values are specified (in Table 2), including those from literature [23–25] as follows:

**Table 2:** Parameters values and references.

Parameter	Description	Value	Range	Reference
Λ	Recruitment rate of the susceptible *S*	14.63	[1,50]	Assumed
μ	Natural exit rate of all individuals	0.000042	[0,1]	Estimated
η	Effective contact rate of *S* or $S_{p}$ with *I*	0.35	[0.3,1]	[11,12]
$\eta_{p}$	Effective contact rate of *S* or $S_{p}$ with $I_{p}$	0.20	[0.2,0.85]	[11,12]
$\eta_{a}$	Effective contact rate of *S* or $S_{p}$ with *A*	1.33	[0.2,1.5]	[11,12]
$\eta_{1}$	Effective contact rate of *S* or $S_{p}$ with *M*_1_	0.0022	[0,1]	Assumed
$\eta_{2}$	Effective contact rate of *S* or $S_{p}$ with *M*_2_	0.0018	[0,1]	Assumed
$\varepsilon_{p}$	Efficacy level of e.s.m awareness	0.8	[0,1]	By definition
$\varepsilon_{r}$	Social Media accessibility regulation rate	0.65	[0,1]	By definition
γ	Enrolment rate into e.s.m awareness	0.35	[0,1]	By definition
θ	Contribution of $M_{k}$ growth by *I*	0.0024	[0.0020,0.0050]	Assumed
$\theta_{a}$	Contribution of $M_{k}$ growth by *A*	0.0038	[0.35,0.0080]	Assumed
$\rho_{1}$	Progression rate from *I* to *A*	0.26	[0.18,0.30]	[11,22]
$\rho_{2}$	Progression rate from $I_{p}$ to *A*	0.39	[0.18,0.9]	[11,23,24]
$\delta_{1}$	Exclusion rate in *I* due to ${ℳ}_{k}$ use	0.010	[0.01, 0.05]	Assumed
$\delta_{2}$	Exclusion rate in $I_{p}$ due to ${ℳ}_{k}$ use	0.020	[0.01, 0.05]	Assumed
$\delta_{3}$	Exclusion rate in *A* due to ${ℳ}_{k}$ use	0.045	[0.01, 0.05]	Assumed
$\sigma_{1}$	Natural decay rate of *M*_1_	0.024	[0.02,0.03]	Assumed
$\sigma_{2}$	Natural decay rate of *M*_2_	0.015	[0.01,0.02]	Assumed


$$\begin{array}{c} S(0)=480,\;S_{p}(0)=12,\;I(0)=4,\;I_{p}(0)=0,\;A(0)=4,\;M_{1}(0)=10^{5},\;M_{2}(0)=10^{3}. \end{array}$$

### Sensitivity analysis of parameters on ${ℛ}_{c}$

To address the uncertainty in the parameter values assumed, which may result into poor understanding and compromising outcomes, we conduct a partial rank correlation coefficients (PRCCs) method on the control reproduction number ${ℛ}_{c}$. PRCCs method is a robust statistical method that quantifies the strength and direction of the relationship between each parameter and the model output. We use of the spearmanr function in Python module scipy.stats. The PRCCs simulation results are presented in Fig 4.

**Fig 4:**
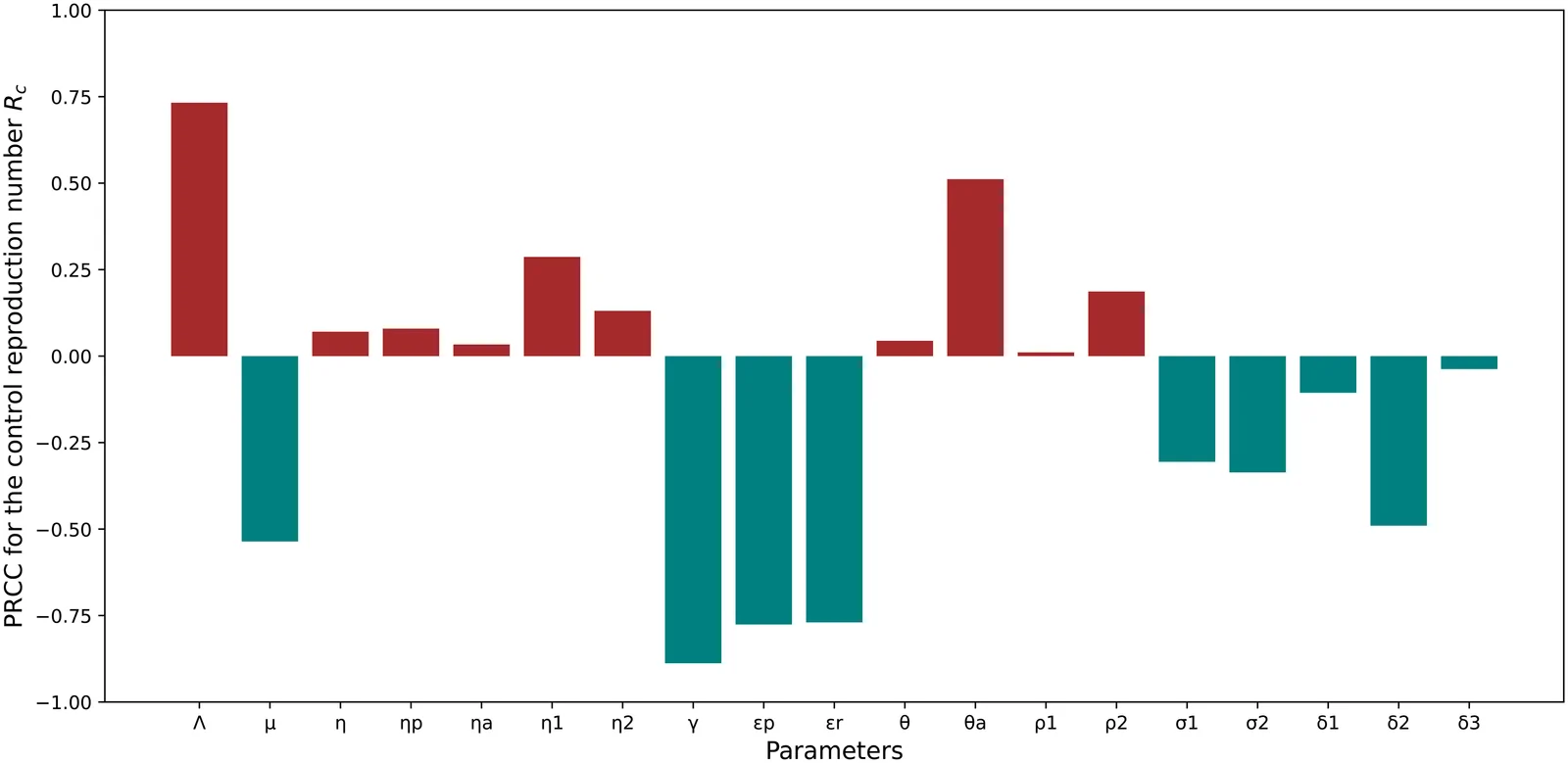
Partial rank correlation coefficients illustrating the effect of each model parameter’s variations on ${ℛ}_{c}$.

Parameters with positive PRCCs (above the horizontal axe) would contribute into the persistence of the social media addiction transmission, while those with negative PRCCs (below the horizontal axe) will help hindering the spread of the harmful impact of social media excessive use, whenever their value increases, respectively.

The results point out all the transmission contact rates (η, $\eta_{p}$, $\eta_{a}$, $\eta_{1}$, and $\eta_{2}$) as well as the contribution of the infected/addicted students into the pathogenic content growth (θ, $\theta_{a}$) as transmission drivers. This translates that student behaviours associated to these parameters would trigger the dynamics of the excessive use of the social medial within the student population.

On the other hand, parameters with negative PRCCS including the awareness rate (γ), its efficacy level $\varepsilon_{p}$, and the accessibility regulation rate $\varepsilon_{r}$, and the remaining parameters are shown to be impeding parameters. This later thus highlights the essential need of reinforcing the social media awareness enrolment rate, and this at a high efficacy level, alongside strong monitoring and regulation of the social media platforms accessibility.

We shall then investigate the variation of the infected and the addicted populations, should these key control parameter values ($\gamma ,\varepsilon_{p},\varepsilon_{r}$) increase subsequently in a scenario of combining interventions strategy or a single strategy through numerical simulations.

In addition, the normalized forward sensitivity indices $\left(\Upsilon_{p}^{{ℛ}_{c}}=\frac{\partial {ℛ}_{c}}{\partial p}\times \frac{p}{{ℛ}_{c}}\right)$ for the critical parameters governing our system relative to the control reproduction number (${ℛ}_{c}$) are derived. This is for tracking how changes in peer interactions $\left(\eta ,\eta_{p},\eta_{a}\right)$, awareness program efficacy $\left(\varepsilon_{p}\right)$, and accessibility regulations $\left(\varepsilon_{r}\right)$ to social media platforms scale the epidemic threshold. The results are summarized in Table 3.

**Table 3:** Normalized Forward Sensitivity Indices of ${ℛ}_{c}$ against model key parameters.

Parameter	η	$\eta_{p}$	$\eta_{a}$	γ	$\varepsilon_{p}$	$\varepsilon_{r}$
Sensitivity index ($\Upsilon_{p}^{{ℛ}_{c}}$)	0.83	1.28	1.53	− 0.54	− 0.13	−2.98

The negative indices indicate that increasing these control parameters reduces ${ℛ}_{c}$ while those with positive index values increase ${ℛ}_{c}$ as their value increases. The results quantitatively confirm the numerical simulations in Fig 4 showing that strengthening accessibility regulation and awareness programmes are effective strategies for reducing excessive social-media use among students. Moreover, the results indicate that in a scenario considering γ=35%, $\varepsilon_{p}=85\%$ and $\varepsilon_{r}=75\%$, a 1% increase in $\varepsilon_{r}$ produces approximatively a 2.98% decrease in number of newly addicted students, while a 1% increase in enrolling new students into social media awareness program decrease ${ℛ}_{c}$ by about 0.54%. The awareness campaigns is also showed to be very beneficial in decreasing the social media harmful impact. A 1% increase in awareness efficacy level subsequently reduces the new addiction tendency among the students by about 0.13%.

### Numerical simulations: Control intervention strategies and interpretations

We now undertake simulations of the model using the parameter values gathering in Table 2 to quantify the model qualitative results. Some parameter values in Table 2 have been calibrated based on empirical prevalence data and behavioural compartmental literature in [11,12,21,25]. We explore the impact of the variation of the key control parameters γ, $\varepsilon_{p}$, and $\varepsilon_{r}$ on the infected sub-populations *I*(*t*), $I_{p}(t)$, and *A*(*t*). The results are presented in Figs 5–Fig 6, Fig 7, Fig 8 and interpretation are given in the sections below.

**Fig 5:**
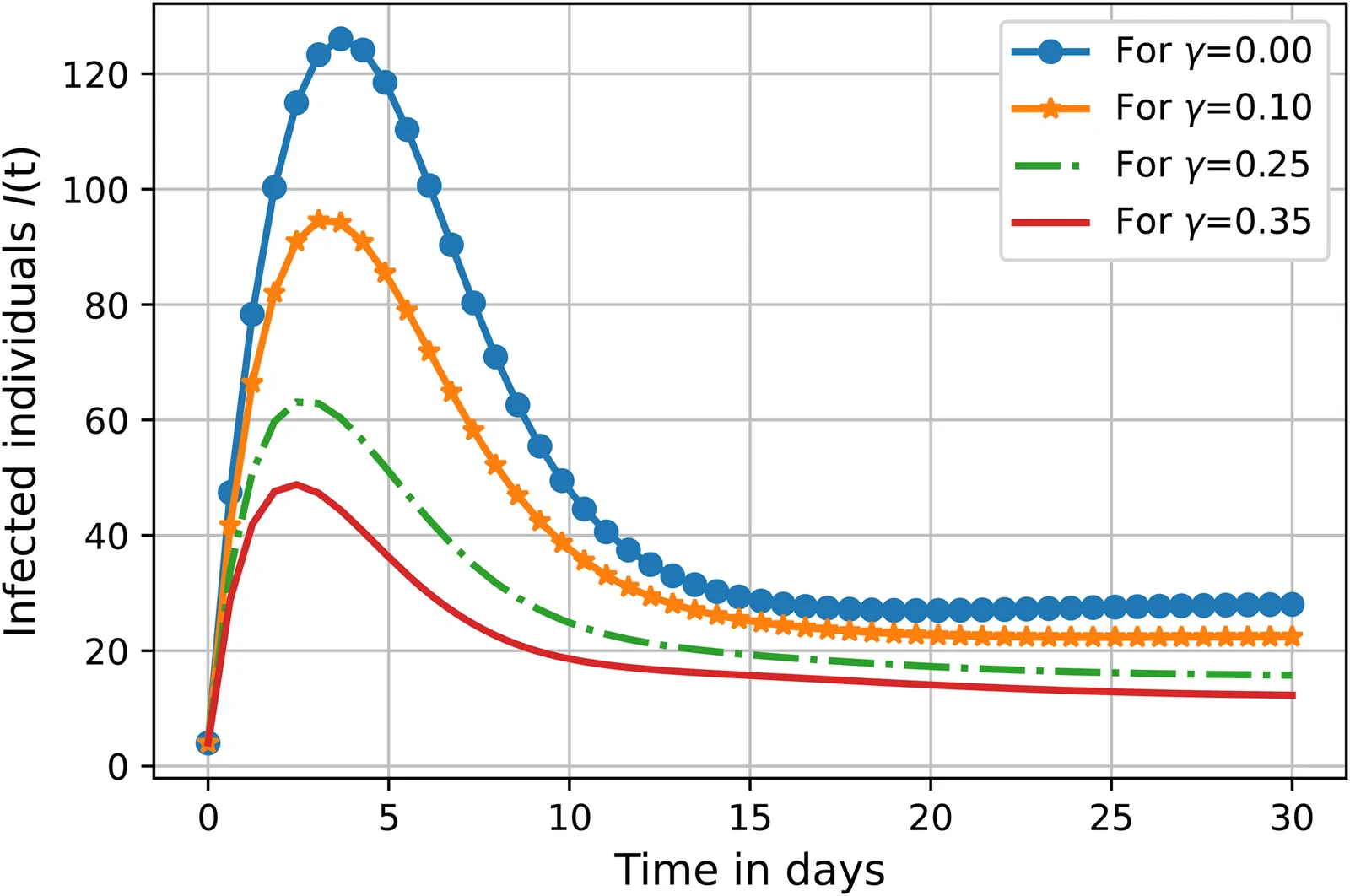
Impact of awareness enrolment rate (γ). Simulations of the infected comportment *I*(*t*) for different levels of awareness enrolment γ = 0.00, 0.10, 0.25, and 0.35. All other parame*t*ers are fixed at the baseline values given in Table 2. The results show that increasing awareness enrolment substantially reduces the peak number of infected students.

**Fig 6:**
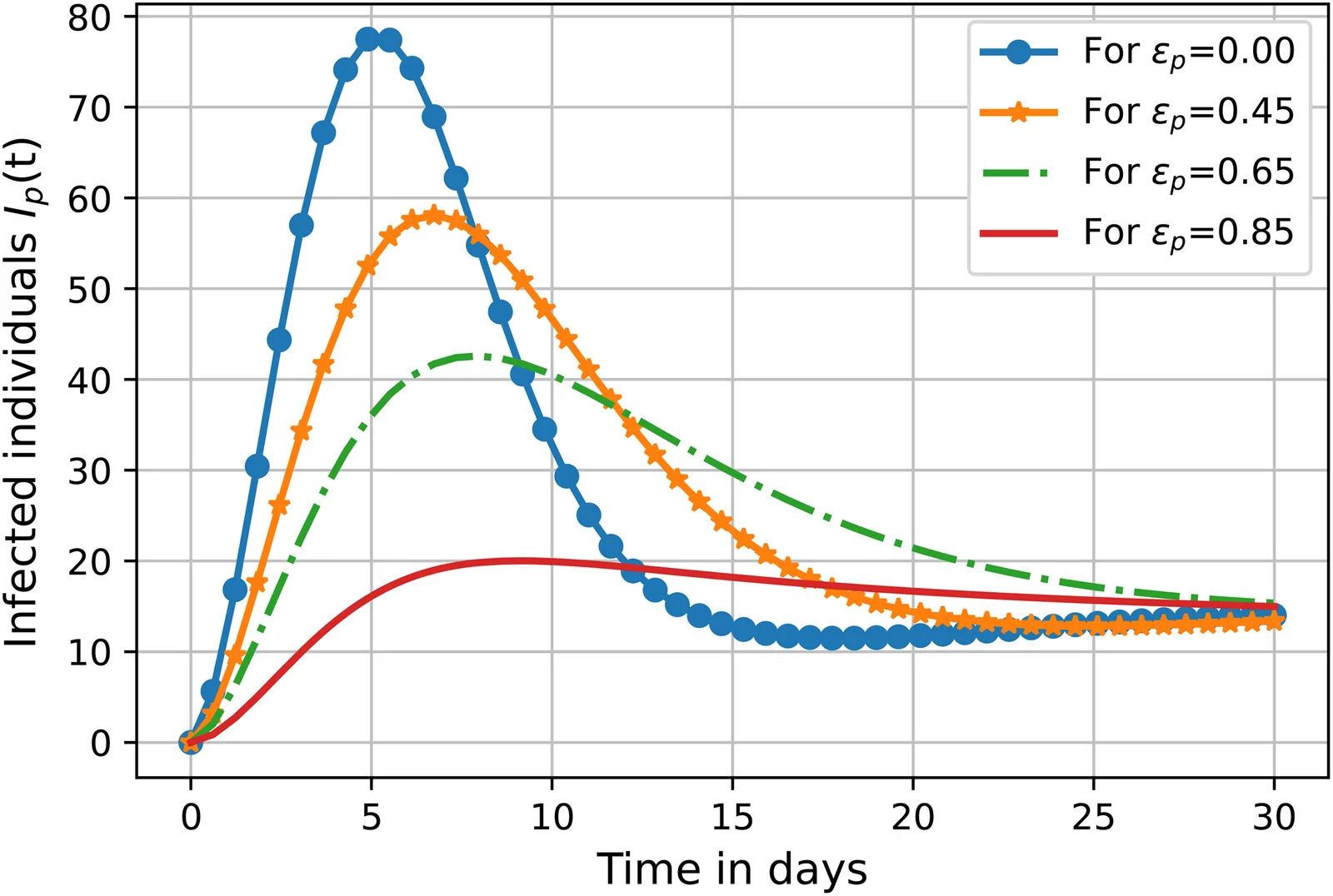
Impact of awareness programme efficacy ($\varepsilon_{p}$). Simulations of infected comportment $I_{p}(t)$ for increasing awareness efficacy levels $\varepsilon_{p}$ = 0.00, 0.45, 0.65, and 0.85. All remaining parameters were maintained fixed at their baseline values listed in Table 2. Higher effectiveness of awareness programmes reduces the number of students becoming infected through awareness failure.

**Fig 7:**
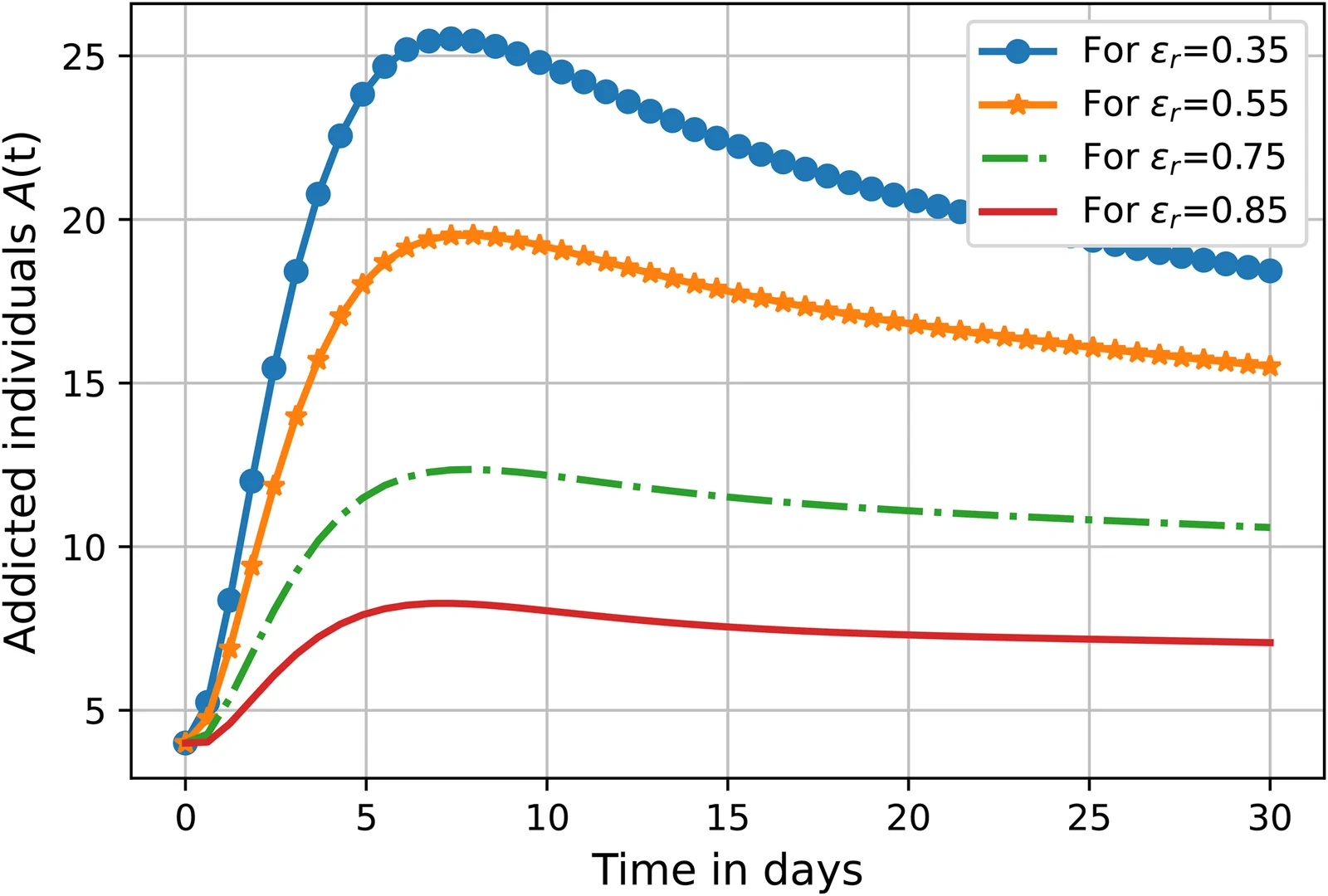
Impact of social media accessibility regulation ($\varepsilon_{r}$) on the addicted student population. Simulations of the addicted compartment *A*(*t*) for different levels of accessibility regulation $\varepsilon_{r}$ = 0.35, 0.55, 0.75, and 0.85. Parame*t*er values are taken from Table 2. Increasing accessibility regulation markedly reduces the number of addicted students and weakens addiction persistence.

**Fig 8:**
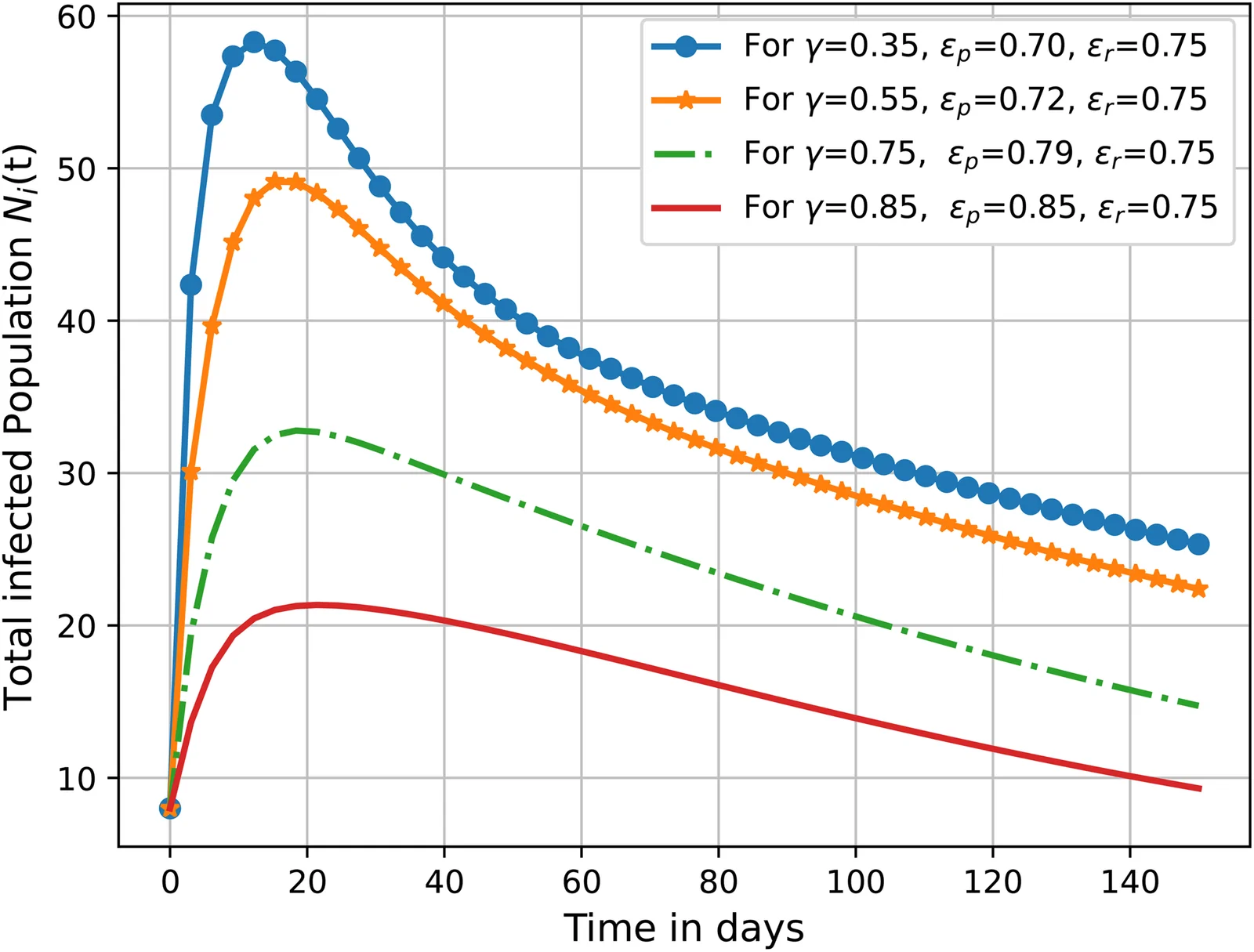
Combined effects of awareness and accessibility-control interventions. Simulations of the total infected population $N_{i}(t)=I(t)+I_{p}(t)+A(t)$ under increasing awareness enrolment and efficacy ($\gamma ,\varepsilon_{p}$) while maintaining a moderate accessibility regulation level ($\varepsilon_{r}=0.75$). The strategy combinations considered are $\left(\gamma ,\varepsilon_{p}\right)$ = (0.35,0.70), (0.55,0.72), (0.75,0.79), and (0.85,0.85). Parameter values are held fixed and taken from Table 2. The combined strategy drives the system from a persistent addiction state toward eradication (${ℛ}_{c}<1$).

#### Strategy 1: Increasing awareness intervention (γ).

We explore the impact of improving awareness enrolment rate (γ) from a poor situation, say γ=2% to an improved level, for instance, γ=35%, targeting the newly registered students (the naive susceptible) for enrolment. This would consist of introducing mandatory digital literacy workshops and seminars, launching students as social media ambassadorship empowerment including senior students and alumni to educate their peers on the possible harms of social media, also inviting higher learning education experts to give talks on the impact of social media on mental health and academic performances, initiate events like screen-free days or digital detox challenges to encourage students to take a break from social media and reflect on their usage.

The numerical results of introducing such interventions are presented in Fig 5.

The results show that at a poor level of awareness enrolment rate (γ=2%), the number of newly distracted students (infected) would raise from an initial value *I*(0)=4 to $I^{**}=100$ active subscribers, within the first four weeks, whereas improving such petty level to a moderate (γ=35%) would bring the corresponding alarming peak value down to $I^{**}=45$. A record of $\Delta I^{**}\approx 50\%$ reduction in newly active subscribers. The intervention has thus helped steer students away from social media nuisance. This emphasizes the benefit and the need of incorporating social media harmful effects awareness campaigns into students’ academic development strategies. This would surely demand a consistent efforts to maintain a high awareness efficacy level. We next investigate the input of efficacy level parameter.

#### Strategy 2: Optimizing the efficacy level of the awareness ($\varepsilon_{p}$).

We then investigate the impact of optimizing the efficacy level $\varepsilon_{p}$ of the awareness campaigns. The aim and objective of this intervention are to promote positive change and effective impact of the efforts of awareness campaigns. This would consist of incorporating within the institution’s premises, eye-catching visual contents such as info-graphics or videos, convey complex information in an engaging and shareable format, utilize various channels, including emails and in-person events, reach different segments of the students, use data and research findings (e.g., this study results) to increase awareness efforts, also establish peer support groups, where students can share their experiences and learn from one another, offer time to time booster sessions to keep the students updated, continuously evaluate and refine awareness efforts to ensure they remain effective and relevant, and track progress and evaluate effectiveness.

This intervention prevents the already aware and enrolled students from reverting to harmful habits of frequent social media use. We thus subsequently increase the values of $\varepsilon_{p}$ from 0% to 85%. The model simulations outputs are presented in Fig 6.

We observed a significant and better decrease in the peak values of the number of infected students due to awareness failure $I_{p}^{*}$. A scenario of efficacy level at $\varepsilon_{p}=85\%$ would reduce the peak value $I_{p}^{*}=60$ corresponding to $\varepsilon_{p}=45\%$, to $I_{p}^{*}=20$, in the first weeks. This, however, tends and stabilizes at the persistent addiction steady state (the red curve); as the new infection rate ${ℛ}_{c}$ remains above the unit.

In addition, a trajectory crossover (switching) in $I_{p}(t)$ is observed between *t* = 15 and *t* = 20 days. This can be dynamically explained by the fact *t*ha*t* for low awareness efficacy rate $\varepsilon_{p}=0\%$ (blue curve), an unchecked epidemic peak rapidly depletes the susceptible students population, causing a subsequent steep decline in $I_{p}(t)$ due to rapid progression $\left(\rho_{2}\right)$ to addiction and institutional exclusion ($\delta_{2}$). Conversely, higher efficacy $\varepsilon_{p}=85\%$ cushion the initial outbreak, maintaining a larger reservoir of uninfected students. This produces a smoother, prolonged infection curve that temporarily exceeds the rapidly falling low-efficacy curve (blue curve) before both settle into the persistent addiction equilibrium point ${ℰ}^{*}$, corresponding to infection rate ${ℛ}_{c}=1.58>1$.

This tells that raising the awareness at its optimal efficacy level ($\varepsilon_{p}=85\%$) might be insufficient in reaching full blown eradication. Diligent efforts can be directed to social media sites accessibility regulation. We then explore accessibility regulation interventions and its combination with effective awareness at their higher levels.

#### Strategy 3: Reinforcing social media accessibility regulation policies ($\varepsilon_{r}$).

This strategy is primarily linked to limiting on campus Wi-Fi access to social media sites during study times. This might be implemented by setting boundaries for social media use on academics premises such as libraries and lecture venues, provide educational knowledge on symptoms related to addiction, implement guidelines for responsible social media use, and offer stress and depression management counselling to those who choose to quit social media excessive use. These policies can help promote a healthier and productive social media addiction free environment for the students, boosting their academic success and well-being.

We thus consider different scenarios increasing the value of the parameter $\varepsilon_{r}$ from a low level to high level; we take $\varepsilon_{r}$= 35%,...,85%. The results presented in Fig 7 promise a drastic decrease in number of the highly addicted users. The results thus demand an optimal level of accessibility regulation beyond 85% during the students study time to detox the existing academic environment.

Nevertheless, it is worth mentioning that depriving modern youth of social media access, at such a minimum level $\varepsilon_{r}^{min}=85\%$, might raise objections and lead to claims of digital accessibility rights violation and consequently campus strikes. We then investigate a combined strategy where awareness and accessibility regulation are concurrently implemented.

#### Strategy 4: Combining both awareness programs and accessibility regulation policies concurrently ($\gamma ,\varepsilon_{r}$).

We consider a moderate accessibility control rate ($\varepsilon_{r}=75\%$) coupled with a progressively increase of enrolment rate (γ≥35%) and a efficacy level beyond ($\varepsilon_{p}=70\%$). The results presented in Fig 8 demonstrate a significant decrease, after the first 21 days (3 weeks time), in social media platforms overuse habits yielding a total eradication. This approach moves the infection dynamics from an endemic state (${ℛ}_{c}>1$) to a total eradication state (${ℛ}_{c}<1$), which corroborate with the stability Theorems 0.3, 0.4. Hence, this strategy’s results can contribute to significant student development policies and therefore promote better academic performance from the distracted students.

## Model findings and practical applicability for higher education leaders

To maximize the practical translation of this seven-compartment model into strategic institutional governance, our mathematical pathways are mapped directly onto actionable policy levers. By converting abstract parameter values into concrete administrative interventions, university leaders can interpret the model results through the two primary operational dimensions as follows:

- **Proactive prevention and compliance metrics (**γ**,** $\varepsilon_{p}$**):** The mathematical transition of naive susceptible students in compartment *S*(*t*) into the protected aware compartment $S_{p}(t)$ is governed by the enrolmen*t* rate γ and its implementation efficacy level $\varepsilon_{p}$. In a non-mathematical context, the model findings highlights that introducing digital wellness campaigns (γ) is insufficient if the message fails to resonate and reached target students. University leaders must treat $\varepsilon_{p}$ as a institutional benchmark for engagement, measuring the quality, absorption, and behavioural impact of mandatory freshman orientation workshops and structured campus-wide digital detox challenges through visual signs. The model thus confirms that maximizing $\varepsilon_{p}$ actively shrinks the downstream “infection” pathways before students develop chronic academic impairments.
- **Structural environmental interventions (**$\varepsilon_{r}$**):** The model incorporates the parameter $\varepsilon_{r}$ to represent an external, structural control on direct social media addictive contents exposure ($M_{k}$). For teaching and learning officers and students academic wellness leaders, this represents technical infrastructure policies rather than behavioral appeals. Implementing social media accessibility restrictions $\varepsilon_{r}$ translates to the strategic blocking, network throttling, or bandwidth-capping of high-traffic, and non-academic media endpoints on institutional Wi-Fi networks. Rather than deploying blanket campus censorship, the model supports a localized strategy: enforcing high network constraints ($\varepsilon_{r}$) within dedicated academic sanctuaries such as lecture halls, laboratories, and core library spaces during peak instructional hours, while maintaining unhindered access in residential and recreational spaces.

The model findings suggest that successfully implementing the above items will reduce the control reproduction number ${ℛ}_{c}$ below unit, thereby confirms a total eradication of social media addiction within the student population. Ultimately, these aforementioned items demonstrate that the control reproduction number (${ℛ}_{c}$) is not be regarded as an abstract threshold, but a decision dashboard tool. In addition, our model promise that by systematically increasing proactive education ($\gamma ,\varepsilon_{p}$) and balancing structural social media regulations within the university ($\varepsilon_{r}$), the university administrations possess a mathematically validated blueprint to suppress campus-wide digital contagion, improve academic performances, and protect institutional academic integrity.

## Discussion

This study was motivated by the prevalent degrading and addictive habits of excessive social media use observed among the majority of higher education learners, which often result in poor academic performance and therefore trigger increased drop-out rates. We thus formulated a mathematical model that partitions a population of students into susceptible, active (infected), and addicted students using mathematical modelling. The addiction transmission dynamics are governed by the interactions between the susceptible students and typical distracted students and direct contract with social media contents. The study proposes awareness campaigns and reinforcement of social media accessibility policies within each institution as control interventions to address these problems. The model’s qualitative analyses were rigorously undertaken, including positivity and boundedness of the model solutions. The model’s equilibrium points and stability analyses inform that the model undergoes a forward transcritical bifurcation; a phenomenon that guaranteeing that maintaining the number of new students developing social media addiction habits below one (${ℛ}_{c}<1$) would be sufficient to detoxify the active/addictive students from the addiction and purify the learning atmosphere. The model simulation results validate the qualitative results and indicate that both control interventions, when consider either as a single strategy, would be beneficial in averting new students’ indulgence into such habits but may demand more effort to refocus the already distracted students. A combined strategy of both control interventions at optimal efficacy levels promise a healthier academic environment and improving overall student performance.

Our findings are consistent with those reported by Ali *et al.*[11] and Alemneh and Alemu in [12] who demonstrated that awareness-based interventions can mitigate the spread of social media addiction. However, unlike Ali *et al.*[11], who modelled social media addiction primarily through homogeneous interpersonal interactions, the present study explicitly distinguishes between peer-to-peer transmission and direct student-to-media transmission through addictive online contents. In addition, while Alemneh and Alemu [12] incorporated awareness and control measures, our model formulation further separates students enrolled in awareness programmes ($S_{p}$) from those not enrolled and introduces the awareness-failure compartment ($I_{p}$), allowing the effectiveness of awareness campaigns to be quantified more realistically. The inclusion of the media-content compartments ($M_{k},\;k=1,2$) provides an explicit representation of the role of addictive digital content in sustaining social media overuse. Consequently, the model offers new insights into how institutional awareness programmes and accessibility regulations interact with both social influences and direct exposure to online content, thereby providing a more comprehensive framework for evaluating intervention strategies in higher education settings.

## Conclusion

This study demonstrates that effective regulation and awareness strategies are critical in mitigating the negative impact of excessive social media use on academic performance. A strict monitoring of the social media platforms accessibility concurrently implemented with awareness enrolment and its efficacy at high levels remains the best strategy in preventing new harmful social media habits and averting addiction development trends. The model findings thus highlight several important implications for higher education institutions. The sensitivity and simulation analyses identify peer influence, direct exposure to addictive social media content, and content generation by active users as the primary drivers of excessive social media use among students. Conversely, awareness enrolment, awareness programme efficacy, and accessibility regulation are shown to be effective control measures that reduce the spread of harmful social media use. These results suggest that universities should invest in early digital-wellness education, strengthen the quality and reach of awareness campaigns, and implement balanced accessibility-control policies within learning spaces. While restrictions on social media access must be carefully designed to respect students’ autonomy and digital rights, targeted regulation in academic environments may help maintain student engagement and academic focus. The study thus contributes a dual-transmission framework that captures both peer influence and direct exposure to addictive social media content within higher education institutions.

Overall, the model provides a quantitative framework that can support institutional decision-making aimed at promoting healthier digital habits and improving academic performance.

However, a limitation of the present study is that the model has not yet been calibrated or validated using empirical data from a specific higher education institution. The current work is intended as a theoretical framework that provides qualitative insights into the dynamics of excessive social media use and the potential effectiveness of intervention strategies. Future research will focus on collecting institutional survey or longitudinal data to estimate model parameters, validate the model predictions, and evaluate the proposed interventions under real-world conditions. This will enhance the model’s predictive capability and support evidence-based policy recommendations.

## Appendix

The defined function ϕ is continuous, positive, and decreasing, given by


$$\begin{array}{cc} \phi & :\Omega ⟶{\mathbb{R}}_{+}\\ & \lambda ⟼\alpha_{1}\frac{1}{f_{1}\lambda +\mu }+\alpha_{2}\frac{1}{\left(f_{4}+f_{3}\lambda )(f_{1}\lambda +\mu \right)}, \end{array}$$

Where $\alpha_{1}$ and $\alpha_{2}$ are positive and composed of the model parameters, so that, 𝒵(λ)=1−ϕ(λ) is bounded below by $1-{ℛ}_{c}$ and above by 1.

## Supporting information

S1 File Campus data. (DOCX)

## References

[1] Anderson M, Jiang J. Teens, technology and friendships. Washington (DC): Pew Research Center; 2018. Available from: https://www.pewresearch.org/internet/2018/11/28/teens-social-media-habits-and-experiences/

[2] Rideout VJ, Robb MB. The Common Sense Census: Media Use by Tweens and Teens. San Francisco (CA): Common Sense Media; 2022.

[3] Statista. Number of mobile social media users worldwide from 2017 to 2028. Hamburg: Statista; 2024. Available from: https://www.statista.com/

[4] Statista. Most popular social networks worldwide as of January 2024, ranked by number of monthly active users. Hamburg: Statista; 2024. Available from: https://www.statista.com/

[5] Kirschner PA, Karpinski AC. Facebook® and academic performance. Comput Hum Behav. 2010;26(6):1237–45. doi: 10.1016/j.chb.2010.03.024

[6] Kuss DJ, Griffiths MD. Social Networking Sites and Addiction: Ten Lessons Learned. Int J Environ Res Public Health. 2017;14(3):311. doi: 10.3390/ijerph14030311 28304359 PMC5369147

[7] M. Talaue G, AlSaad A, AlRushaidan N, AlHugail A, AlFahhad S. The Impact of Social Media on Academic Performance of Selected College Students. IJAIT. 2018;8(4/5):27–35. doi: 10.5121/ijait.2018.8503

[8] Kuss DJ, Griffiths MD. Online social networking and addiction--a review of the psychological literature. Int J Environ Res Public Health. 2011;8(9):3528–52. doi: 10.3390/ijerph8093528 22016701 PMC3194102

[9] Villagrá-Arnedo CJ, Gallego-Durán FJ, Llorens-Largo F, Compañ-Rosique P, Satorre-Cuerda R, Molina-Carmona R. Improving the expressiveness of black-box models for predicting student performance. Comput Hum Behav. 2017;72:621–31. doi: 10.1016/j.chb.2016.09.001

[10] Briz-Ponce L, Pereira A, Carvalho L, Juanes-Méndez JA, García-Peñalvo FJ. Learning with mobile technologies–students’ behavior. Comput Hum Behav. 2017;72:612–20.

[11] Ali AS, Javeed S, Faiz Z, Baleanu D. Mathematical modelling, analysis and numerical simulation of social media addiction and depression. PLoS One. 2024;19(3):e0293807. doi: 10.1371/journal.pone.0293807 38470872 PMC10931467

[12] Alemneh HT, Alemu NY. Mathematical modeling with optimal control analysis of social media addiction. Infect Dis Model. 2021;6:405–19. doi: 10.1016/j.idm.2021.01.011 33615084 PMC7881229

[13] Abi MM, Bano EN, Obe LF, Blegur FM. Mathematical modeling and simulation of social media addiction TikTok using SEI1I2R type model. J Diferensial. 2023;5(1):43–55.

[14] Bansal K, Mathur T, Mathur T, Agarwal S, Sharma RD. Impact of social media on academics: a fractional order mathematical model. Int J Model Simul. 2025;45(4):1410–24. doi: 10.1080/02286203.2023.2286419

[15] van den Driessche P, Watmough J. Reproduction numbers and sub-threshold endemic equilibria for compartmental models of disease transmission. Math Biosci. 2002;180:29–48. doi: 10.1016/s0025-5564(02)00108-6 12387915

[16] Dushoff J, Huang W, Castillo-Chavez C. Backwards bifurcations and catastrophe in simple models of fatal diseases. J Math Biol. 1998;36(3):227–48. doi: 10.1007/s002850050099 9528119

[17] Lakshmikantham V, Leela S, Martynyuk AA. Stability analysis of nonlinear systems. New York: M. Dekker; 1989.

[18] Gumel AB. Causes of backward bifurcations in some epidemiological models. J Math Anal Appl. 2012;395(1):355–65. doi: 10.1016/j.jmaa.2012.04.077

[19] Castillo-Chavez C, Song B. Dynamical models of tuberculosis and their applications. Math Biosci Eng. 2004;1(2):361. 10.3934/mbe.2004.1.361 20369977

[20] 2025 Mathematics and Mathematics Education student enrolment at the University of the Free State, QwaQwa Campus, South Africa. 10.5281/zenodo.22111219

[21] Salari N, Zarei H, Hosseinian-Far A, Rasoulpoor S, Shohaimi S, Mohammadi M. The global prevalence of social media addiction among university students: a systematic review and meta-analysis. J Public Health (Berl). 2025;33(1):223–36. doi: 10.1007/s10389-023-02012-1

[22] Guo Y, Li T. Optimal control and stability analysis of an online game addiction model with two stages. Math Meth Appl Sci. 2020. doi: 10.1002/mma.6200

[23] Andrawus J, Iliyasu Muhammad A, Akawu Denue B, Abdul H, Yusuf A, Salahshour S. Unraveling the importance of early awareness strategy on the dynamics of drug addiction using mathematical modeling approach. Chaos. 2024;34(8). doi: 10.1063/5.0203892 39141788

[24] Saidi Kalula A, Nyabadza F. A theoretical model for substance abuse in the presence of treatment. S Afr J Sci. 2012;108(3/4). doi: 10.4102/sajs.v108i3/4.654

[25] Andreassen CS, Billieux J, Griffiths MD, Kuss DJ, Demetrovics Z, Mazzoni E, et al. The relationship between addictive use of social media and video games and symptoms of psychiatric disorders: A large-scale cross-sectional study. Psychol Addict Behav. 2016;30(2):252–62. doi: 10.1037/adb0000160 26999354

