# Supplementary material for: Impact of social media platforms excessive use on students performance in higher education institutions: A mathematical model approach
Source: PLoS One. 2026 Oct 1;21(10):e0359301. doi: 10.1371/journal.pone.0359301 (PMC13630262; doi:10.1371/journal.pone.0359301)
Supplement: Supplementary file 2 [file pone.0359301.s001.docx]

| Campus | QWA |  |  |  |  |  |
| --- | --- | --- | --- | --- | --- | --- |
|  |  |  |  |  |  |  |
|  |  |  |  |  |  |  |
| **Mod Enrl** |  |  | **Year** |  |  |  |
| **Module Code** | **Module Name** | **Registered Faculty** | **2022** | **2023** | **2024** | **2025** |
| **MATM1534** | **Calculus** | EDUCATION | 71 | 59 | 138 | 110 |
|  |  | NATURAL AND AGRICULTURAL SCIENCES | 143 | 175 | 214 | 221 |
| **MATM1534 Total** |  |  | **214** | **234** | **352** | **331** |
| **MATM1622** | **Introduction to Advanced Maths** | EDUCATION | 36 | 11 | 29 | 45 |
|  |  | NATURAL AND AGRICULTURAL SCIENCES | 15 | 10 | 3 | 29 |
| **MATM1622 Total** |  |  | **51** | **21** | **32** | **74** |
| **MATM2614** | **Vector Analysis** | EDUCATION | 1 | 2 | 1 | 19 |
|  |  | NATURAL AND AGRICULTURAL SCIENCES | 16 | 9 | 10 | 12 |
| **MATM2614 Total** |  |  | **17** | **11** | **11** | **31** |
| **MATM2664** | **Sequences and Series** | EDUCATION | 7 | 15 | 15 | 17 |
| 0B | 1B | 2BNATURAL AND AGRICULTURAL SCIENCES | 3B10 | 4B9 | 5B7 | 6B11 |
| **MATM2664 Total** |  |  | **17** | **24** | **22** | **28** |
| **MATA2654** | **Ordinary Differential Equation** | EDUCATION | 12 | 18 | 19 | 10 |
|  |  | NATURAL AND AGRICULTURAL SCIENCES | 35 | 19 | 7 | 14 |
| **MATA2654 Total** |  |  | **47** | **37** | **26** | **24** |
| **Grand Total** |  |  | **346** | **327** | **443** | **488** |

DOI: [10.5061/dryad.bzkh189sn](https://doi.org/10.5061/dryad.bzkh189sn)
