# Supplementary material for: Impact of social media platforms excessive use on students performance in higher education institutions: A mathematical model approach
Source: PLoS One. 2026 Oct 1;21(10):e0359301. doi: 10.1371/journal.pone.0359301 (PMC13630262; doi:10.1371/journal.pone.0359301)
Supplement: Supplementary file 10002 [file pone.0359301.s002.pdf]

Date: August 12, 2026

Journal: *PLOS ONE*
Manuscript Title: Impact of social media platforms excessive use on students' performance in higher education institutions: A mathematical model approach
Authors: Komi Afassinou, Narcisse Roland Loufouma Makala, Ousmane Koutou, Richard O. Ocaya, Maba Boniface Matadi.
Manuscript ID: (PONE-D-26-10052R1)

Dear Editor and Reviewers,

We thank the Editor and the Reviewers for their constructive, insightful, and encouraging feedback on our manuscript. We highly appreciate the recognition of our mathematical treatment as "rigorous and impressive."

We have carefully considered the concerns regarding structural complexity, practical accessibility, and grounding in existing literature. Below is our point-by-point response detailing the extensive revisions made to address these comments. Revised text segments are highlighted in our updated manuscript.

Response to Reviewer #1 Comments

Concern 1: Structural Complexity and Accessibility

Reviewer: "*While the mathematical treatment is rigorous and impressive, the model's structural complexity may inadvertently undermine its accessibility and practical applicability.***"**

Response:
We appreciate this crucial observation. The 7-compartment model was designed to capture specific dual-transmission routes. However, we agree that its administrative and practical utility must be clearly translated for non-mathematical readers, such as university administrators and policymakers.
To bridge this gap without diluting the mathematical rigor, we have made a major section to the manuscript explaining the applicability of the model key findings:

Revised Text in Manuscript

Model findings and practical applicability for higher education leaders (Page 17)
To maximize the practical translation of this seven-compartment model into strategic institutional governance, our mathematical pathways are mapped directly onto actionable policy levers. By converting abstract parameter values into concrete administrative interventions, university leaders can interpret the model results through the two primary operational dimensions as follows:

- **Proactive prevention and compliance metric (γ, εp).**
- **Structural environmental interventions (εr).**

Concern 2: Grounding in Existing Literature

Reviewer *"This is further compounded by a lack of grounding in existing literature; the references provided do not sufficiently contextualize the model within established research or clarify how it builds upon previously published frameworks."*

Response:

We acknowledge that our original introduction did not sufficiently contrast our model with the foundational models in epidemiological and behavioural science.

To resolve this, we expanded our literature review in the **Introduction (Page 2)** . We explicitly integrated citations of core compartmental structures, including social media models (e.g., Alemneh & Alemu, 2021; Ali et al., 2024). We then detailed the specific *mathematical gap* our model fills: the distinction between peer-driven infection and direct algorithmic media temptation.

Revised Text in Manuscript (Page 2)

So far, the majority of the research documented in this field has relied on longitudinal studies and qualitative reviews [7–10]. Nevertheless, Ali et al., in [11], successfully mapped social media use as a homogeneous contagious trend. Similarly, Alemneh and Alemu [12] developed a compartmental model incorporating social media awareness and addiction control strategies. Some few compartmental mathematical models including [13, 14] were also proposed in this regard. However, the authors in these pioneer social media addiction compartmental models widely focused their analysis on single-route peer-to-peer transmission, while overlook the dual nature of transmission, specifically the distinction between peer-to-peer influence and direct student-to-media contact. This underscores a significant gap remaining to be addressed in compartmental mathematical models to social media addiction studies. This article addresses this gap by developing a model that partitions the student population into distinct classes based on their level of platform engagement and susceptibility. By explicitly branching the susceptible and infected channels, the model thus builds upon existing literature by isolating how institutional intervention interacts with direct exposure to additive social media contents.

Concern 3: Comment regarding Sensitivity Analysis:

Reviewer: "The manuscript lacks a sensitivity study of the parameters and statistical analysis of their ranges of variation, which is critical for a model of this nature."

Response:

We fully concur with the reviewer that establishing a sensitivity study and parameter variance framework is an absolute prerequisite for validating a compartmental epidemiological model. We thank the reviewer for identifying this omission.

Revised Text in Manuscript (Page 2)

To resolve this gap comprehensively, we have added a new section to our manuscript In Section Sensitivity analysis of parameters on Rc on **Pages 14-15.** In this new addition, we have:

1. Derived the Analytical Normalized Forward Sensitivity Indices (***Rc***) for the critical parameters governing our system relative to the control reproduction number (***Rc***).

2. Isolated the explicit analytical derivatives for tracking how changes in peer interaction (η, ηp, ηa), awareness program efficacy (γ, εp), and social media accessibility regulations (εr) scale the epidemic threshold.

These additions provide the necessary mathematical verification and substantially strengthen the credibility of our model's policy recommendations. And regarding the statistical analysis of their ranges of variation, without delaying the manuscript, data collecting are undergoing for future research work and collaboration with peers in statistics.

Concern 4: Comment regarding discussion and application of model findings:
Reviewer: *"The discussion section is narrow, offering limited reflection on the broader educational, ethical, or institutional implications of the findings".*

Response:
We completely agree with the reviewer that the practical utility of our mathematical model is heavily enhanced by bridging its abstract thresholds with the real-world governance of higher education environments. We thank the reviewer for highlighting this vital area for expansion.

Revised Text in Manuscript (Page 19)
In the revised manuscript, we have completely overhauled and expanded the **Discussion** section by comparing the present study findings to those reported by peers in their previous works.

Our findings are consistent with those reported by Ali et al. [11] and Alemneh and Alemu in [12] who demonstrated that awareness-based interventions can mitigate the spread of social media addiction. However, unlike Ali et al. [11], who modelled social media addiction primarily through homogeneous interpersonal interactions, the present study explicitly distinguishes between peer-to-peer transmission and direct student-to-media transmission through addictive online contents. In addition, while Alemneh and Alemu [12] incorporated awareness and control measures, our model formulation further separates students enrolled in awareness programmes (Sp) from those not enrolled and introduces the awareness-failure compartment (Ip), allowing the effectiveness of awareness campaigns to be quantified more realistically. The inclusion of the media-content compartments (Mk, k = 1, 2) provides an explicit representation of the role of addictive digital content in sustaining social media overuse. Consequently, the model offers new insights into how institutional awareness programmes and accessibility regulations interact with both social influences and direct exposure to online content, thereby providing a more comprehensive framework for evaluating intervention strategies in higher education settings.

Concern 5 : Comment regarding empirical data

Reviewer: *"The most significant deficiency is the absence of a case study or empirical data to exemplify the model's real-world applicability... without empirical validation, it remains a theoretical proposal for an overly complex model".*

Response:
We sincerely thank the reviewer for this valuable observation. We agree that the inclusion of a case study or empirical data would further strengthen the practical applicability of the proposed model. However, the primary objective of this study was to develop a theoretical mathematical framework that captures the mechanisms underlying excessive social media use and its potential impact on students' academic performance, rather than to produce institution-specific quantitative predictions.

1. At this stage, reliable longitudinal datasets containing the variables required by the model (e.g., transitions between awareness, active use, addiction, and social-media content exposure) are not publicly available, making rigorous parameter estimation and model calibration difficult. Consequently, the numerical simulations were designed to illustrate the qualitative behaviour of the model and to investigate the relative effectiveness of different intervention strategies under plausible parameter assumptions. The manuscript explicitly states that several parameter values are assumed or estimated for this exploratory purpose and that sensitivity analysis was conducted to evaluate the robustness of the model conclusions.

2. To address the reviewer's concern, we have revised the manuscript to explicitly acknowledge this limitation in the **Conclusion** section. We now state that the present work should be regarded as a theoretical proof-of-concept that provides a foundation for future empirical studies. We further indicate that future work will focus on calibrating and validating the model using institutional survey data or longitudinal data collected from higher education institutions, allowing parameter estimation and assessment of the predictive capability of the model.

We humbly take the reviewer's recommendation seriously for our long-term research trajectory: primary data collection initiatives have officially commenced across the authors' home institutions to capture localized institutional shifts. This ongoing data will form the empirical backbone of subsequent specialized statistical extensions for future research work.

Response to Reviewer #2 Comments

Remark 1
Reviewer: *"The work is novel and addressed a great problem faced by young ones in higher institutions of learning."*

Response: We appreciate the reviewer's positive feedback regarding the social and academic novelty of our research.

Remark 2

Reviewer: *"Line 112: Let Positivity if solutions of the model be Theorem 1; Boundedness of the model solutions be Theorem 2; ... in that order".*

Response:

We have corrected the theorem numbering sequence starting at Line 99 (in the revised text). Section (at page 5) now states **Theorem 0.1** for the *Positivity of Solutions*, followed sequentially by **Theorem 0.2** for the *Boundedness of Solutions*. All subsequent mathematical theorems throughout the paper have been re-indexed in proper sequential order.

Remark 3

Reviewer: *"Line 283: To validate (instead of 'to valid')."*

Response:

Corrected. The text at Line 282 (**Page 13** in the revised text) has been modified to read: *"To validate the qualitative results, we undertake numerical simulations..."*

Remark 4

Reviewer: *"Numerical Analysis: What informed the choice of the values of your initial conditions?"*

Response:

We have expanded the opening paragraph of **Section (Numerical Analysis)** to justify our initial conditions. The initial cohort setup (N(0)=500) models a representative group of university students (freshmen, second year, and repeating students) in the corresponding author's institution (University of the Free State) enrolled in mathematics and math-education in year 2025 . Freshmen are initially modelled as naive susceptible (S(0)=480), while baseline configurations for existing infected (I(0)=4, Ip(0)=0) and addicted (A(0)=4) compartments were informed by real-world screen-time usage averages and higher-education retention metrics found in recent behavioural research (Andreassen CS, 2016; Salari N, 2025 & Kuss DJ, 2017).

Refer to: https://doi.org/10.5281/zenodo.22111219

Remark 5

Reviewer: *Line 298: Partial Rank Correlation Coefficient (instead of 'prank rank...').*

Response:

Corrected. We have replaced the typographical error with the full correct terminology: **Partial** Rank Correlation Coefficient (PRCC). (Page 13 in the revised text)

Remark 6

Reviewer: *Table 2: Most of the parameter values were assumed. What are the basis for assumptions of values?*

Response:
We thank the reviewer for this important observation. We have revised Table 2 to provide clearer justification for the assumed parameter values used in the numerical simulations. Since empirical estimates for several model parameters are currently unavailable in the social media addiction literature, especially for the newly introduced transmission pathways and control-related parameters, baseline values were selected to ensure mathematical and behavioural consistency with the model assumptions. Specifically, the assumed effective contact rates were chosen to reflect the relative influence of the different categories of users. Thus, the parameter values satisfy the hierarchy $0 < n_p < \eta < n_a$, ensuring that addicted individuals exert a stronger behavioural influence on susceptible students than active users, while students infected through awareness failure exert the lowest influence. Similarly, progression, exclusion, content-generation, and content-decay rates were assigned values within realistic ranges that preserve the qualitative dynamics of the system and guarantee epidemiologically meaningful behaviour.

For parameters for which no published estimates currently exist, the values were selected intuitively and conservatively based on the model structure, behavioural interpretation, and consistency with previous compartmental modelling studies where hypothetical baseline values are commonly employed in exploratory investigations. To address the uncertainty associated with these assumptions, we conducted both sensitivity and PRCC analyses, which identify the parameters that most strongly influence the control reproduction number R_c and demonstrate the robustness of the model conclusions to parameter variation.

We have clarified these points in the revised manuscript and explicitly stated that future studies based on empirical and longitudinal data will be necessary to refine these parameter estimates.

Remark 7
Reviewer: *Fig. 5(b): The authors should discuss the reasons for the switching that occurred in the dynamics of the infected individuals, Ip(t) after about 20 days.*

Response:
We thank the reviewer for this perceptive technical observation. We have added a detailed paragraph inside Section to explain this physical behaviour. The initial drop and subsequent "switching" behaviour of Ip(t) around the 20-days mark occurs because optimizing the awareness efficacy (εp) successfully suppresses immediate new failures during the opening weeks. However, because the structural reproduction number Rc remains above unity (Rc=1.58 > 1) due to unchecked student-to-media content growth (Mk), the persistent infection pressures eventually outpace the awareness program, causing the compartment population to pivot back toward the endemic equilibrium state.

1. **Early Phase ($t < 15$ days): Direct Inflow Suppression**
The efficacy level ϵ_p controls the proportion $\left(1-\epsilon_p\right)$ of aware individuals $S_p(t)$ who fail awareness programs and enter the infected class $I_p(t)$. Higher efficacy values ($\epsilon_p = 0.85$) drastically limit the initial rate of new infections. This creates a low, flattened early peak ($I_p^* \approx 20$) compared to low efficacy ($\epsilon_p = 0.00$), where rapid influx causes an early high peak ($I_p^* \approx 78$).

2. **Transition Phase ($t \approx 15-20$ days): Depletion vs. Retention in Susceptible Pool**

   - **Low Efficacy ($\epsilon_p = 0.00$):** High initial transmission rapidly burns through/depleted the available pool of susceptible students. As the supply of uninfected individuals is exhausted and infected individuals progress to addiction ($A(t)$ at rate ρ_2) or exit ($\mu + \delta_2$), $I_p(t)$ undergoes a sharp, steep decline.

   - **High Efficacy ($\epsilon_p = 0.65, 0.85$):** The strong preventive barrier keeps a larger reservoir of susceptible students (S, S_p) uninfected early on. As a result, infections occur at a slower, sustained pace rather than in a sharp single wave, leading to a much gentler decline that temporarily intersects and sits above the rapidly decaying curve of the low-efficacy scenario.

3. **Late Phase ($t > 20$ days): Convergence to the Endemic Equilibrium State**
Because the control reproduction number remains above unity ($\mathcal{R}_c > 1$) in this single-intervention scenario, all curves eventually converge toward the unique Persistent Addiction Equilibrium (PAE) state $\mathcal{E}^*$. The crossover simply reflects two different trajectory paths to the same attractor: one arriving via a high-peak/rapid-burnout curve and the other via a damped, delayed curve.

Remark 8
Reviewer: *Line 332: from a poor situation (instead of 'from a poorly situation').*

Response:
Corrected. The original text at Line 332 now reads: *"...from a poor situation, say (**γ**= 2%) to an improved level..."*. (In the revised text, **page 15**)

Remark 9
Reviewer: *Line 352: Optimizing (instead of 'ptimizing').*

Response:
Corrected. The heading for Section has been fixed to read: *"Strategy 2: Optimizing the efficacy level of the awareness"*. (In the revised text, Line **Page 15**.)

Remark 10
Reviewer: *Lines 398–407: The strategy 4 combined both awareness programs (γ) and accessibility regulation policies (εr) concurrently, which is the goal set at the onset of the study. However, the content of section 4.2.4 (which is also depicted in Fig. 5(d)) showed combination of γ and εp instead. This should be looked into and the graph replotted*

Response:

We sincerely apologize for this glaring structural oversight in our variable labels.

We reviewed our simulation scripts and confirmed that while the computational backend correctly simulated the combined effects of awareness enrolment rate (γ) and accessibility regulation ***(εr)***, the textual descriptions in that Section and labels inside Figure 5(d) erroneously displayed only ***(γ, εp)*** instead of ***(γ, εp, εr)***. We have thoroughly revised Fig 5(d) to firmly emphasize the integration of **(γ)**, ***(εp)***. and ***(εr)***. Figure 5(d) has been updated and replotted with correct legends and parameter markers to maintain total thematic uniformity across the paper.

We believe these adjustments have substantially improved the transparency, positioning, and real-world applicability of our work, matching the high standards of *PLOS ONE*. We hope the manuscript is now acceptable for publication.

Sincerely,

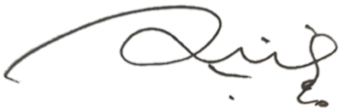

Komi Afassinou
Corresponding Author
Department of Mathematics and Applied Mathematics
University of the Free State, South Africa
Email: Afassinouk@ufs.ac.za / komia@aims.ac.za
